# Multiomics Analyses Reveal an Essential Role of Tryptophan in Treatment of csDMARDs in Rheumatoid Arthritis

**DOI:** 10.1002/advs.202413170

**Published:** 2025-09-23

**Authors:** Congcong Jian, Jing Zhu, Jianhong Wu, Yan Zhang, Jianghua Chen, Huan Wang, Hengyan Liu, Ke Xu, Jiaxin Huang, Xiaoting Zhu, Yuanli Wei, Shilin Li, Tingting Wang, Xuan Huang, Qinghua Zou, Jie Zhang, Jiang Su, Xinming Du, Yaping Lu, Tianci Zhou, Yingtong Zhou, Minglong Tang, Bin Li, Xue Zhou, Qihao Wei, Qiulong Yan, Fanxin Zeng

**Affiliations:** ^1^ Department of Clinical Research Center Sichuan Clinical Research Center for Medical Imaging Dazhou Central Hospital Dazhou 635000 China; ^2^ Medical School Sichuan University of Arts and Sciences Dazhou 635002 China; ^3^ School of Basic Medical Science Chengdu University of Traditional Chinese Medicine Chengdu 611137 China; ^4^ Department of Rheumatology and Immunology Sichuan Provincial People's Hospital Chengdu 610072 China; ^5^ Department of Rheumatology and Immunology Dazhou Central Hospital Dazhou 635000 China; ^6^ Department of Medical Oncology Cancer Center West China Hospital Sichuan University Chengdu 610041 China; ^7^ Dazhou Vocational College of Chinese Medicine Dazhou 635000 China; ^8^ North Sichuan Medical College Nanchong 637100 China; ^9^ Mudanjiang Medical University Mudanjiang 157011 China; ^10^ Medicine School of Henan University Kaifeng 450046 China; ^11^ Institute of Basic Medicine and Forensic Medicine North Sichuan Medical College Nanchong 637100 China; ^12^ Medical Research Center Beijing Chaoyang Hospital Capital Medical University Beijing 100000 China; ^13^ Department of Rheumatology and Immunology First Affiliated Hospital of Army Medical University Chongqing 400038 China; ^14^ Hubei Key Laboratory of Agricultural Bioinformatics College of Informatics Huazhong Agricultural University Wuhan 430070 China; ^15^ Sinopharm (Wuhan) Medical Laboratory Co., Ltd. Wuhan 430000 China; ^16^ Sinopharm (Wuhan) Precision Medical Technology Co., Ltd. Wuhan 430000 China; ^17^ Department of Oncology The Affiliated Hospital of Southwest Medical University Luzhou 646000 China; ^18^ The Fifth Affiliated Hospital of Southern Medical University Guangzhou 510900 China

**Keywords:** csDMARDs, multiomics strategy, rheumatoid arthritis, treatment response, tryptophan

## Abstract

Rheumatoid arthritis (RA) is a autoimmune disease characterized by heterogeneity in response to conventional synthetic disease‐modifying antirheumatic drugs (csDMARDs). This study aims to elucidate molecular differences in response mechanisms of RA patients to csDMARDs through multiomics approach, with particular focus on the role of tryptophan (Trp) in treatment. Plasma, fecal, and peripheral blood mononuclear cells (PBMCs) were collected for metabolomics, microbiomics, transcriptomics, single‐cell transcriptomics, proteomics, and phosphoproteomics analyses. Additionally, in vitro/vivo experiments and controlled clinical trial were conducted to validate the findings. Comprehensive analysis revealed significant alterations in Trp‐related metabolic profiles, microbiota composition, and immune cells, indicating the role of Trp in modulating therapeutic response to csDMARDs. In vitro experiments demonstrated that Trp significantly inhibited the proliferation of MH7A and PBMCs while reducing the secretion of IL‐1β, IL‐6, and TNF‐α. Furthermore, in vivo studies showed that Trp treatment decreased arthritis scores and histological scores in mice. Clinical data further confirmed that dietary supplementation with Trp significantly improved disease activity scores and alleviated inflammatory in RA patients. This study highlights the crucial regulatory role of Trp in RA therapy, providing novel insights for optimizing clinical treatment strategies for RA.

## Graphical abstract

Rheumatoid arthritis is a disease characterized by joint inflammation. Approximately 50% of patients show insufficient response to traditional synthetic disease‐modifying antirheumatic drugs. This study aims to elucidate differential molecular profiles of the mechanisms underlying drug responses through multi‐omics strategy. In vitro/vivo experiments and clinical trial were conducted to verify the role of tryptophan in modulating immune status and drug treatment.

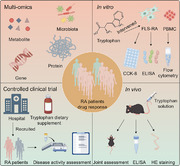

## 1 Introduction

Rheumatoid arthritis (RA) is a typical chronic autoimmune disease primarily characterized by inflammation of the synovial joints, leading to joint swelling, pain, and ultimately resulting in bone destruction, deformity, and dysfunction.^[^ ^1^, ^2^, ^3^ ^]^ According to epidemiological surveys, the prevalence of RA in China ranges from 0.28% to 0.41%, affecting approximately 5 million individuals, with the incidence in women being two to three times higher than that in men.^[^ ^4^, ^5^, ^6^ ^]^ Currently, according to the recommendations of the American College of Rheumatology (ACR) guidelines, conventional synthetic disease‐modifying anti‐rheumatic drugs (csDMARDs) are recommended the first‐line treatment for RA, including methotrexate (MTX), leflunomide (LEF), and hydroxychloroquine (HCQ), which are often combined with non‐steroidal anti‐inflammatory drugs (NSAIDs) and glucocorticoids.^[^ ^7^ ^]^ The ACR70 remission rate in RA patients treated with MTX alone is less than 40%, and nearly 50% of early RA patients achieve remission with combination therapy involving glucocorticoids.^[^ ^1^, ^8^, ^9^ ^]^ Despite continuous modification and optimization of treatment protocols, approximately 50% of patients still do not fully benefit, and the clinical remission rate is far from adequate. However, current studies are still insufficiently exploring the mechanisms of response to csDMARDs therapy in RA patients.

Tryptophan (Trp) is an essential amino acid and an important molecular precursor for protein synthesis and various biological processes that influence pathophysiological conditions, including neurology, immunity, metabolism, inflammation, oxidative stress, and intestinal homeostasis.^[^ ^10^, ^11^ ^]^ Indoleamine 2, 3‐dioxygenase 1 (IDO1) is a key catabolic enzyme that converts Trp into kynurenine to activate aryl hydrocarbon receptor‐induced regulatory T cell (Treg)‐mediated immunomodulation, promoting Treg activation and differentiation to suppress T cell responses.^[^ ^12^, ^13^ ^]^ In autoimmune diseases, enhanced IDO1‐mediated degradation of Trp is regarded as a critical mechanism underlying the dysregulated immune response.^[^ ^14^ ^]^ In inflammatory bowel disease, serum Trp levels are significantly reduced in patients and negatively correlate with C‐reactive protein (CRP), suggesting that Trp deficiency may exacerbate the inflammatory state.^[^ ^15^ ^]^ Additionally, studies have reported significant downregulation of the Trp metabolic profile in the synovium of RA patients, with substantial reductions in serum Trp levels that correlate with disease progression and increased inflammation.^[^ ^16^, ^17^, ^18^ ^]^ Furthermore, clinical evidence suggests that omeprazole combined with Trp significantly promotes the healing of gastroduodenal ulcers.^[^ ^19^ ^]^ However, the role of Trp in csDMARD therapy and related clinical trials in RA remains poorly investigated.

Recent advances in multiomics technologies have significantly enhanced our understanding of RA, particularly regarding its pathogenesis, the discovery of therapeutic targets, and identification of specific biomarkers.^[^ ^20^, ^21^, ^22^, ^23^ ^]^ Several studies have investigated the potential alterations in plasma metabolites, gut microbiota, and genes in RA patients, elucidating the regulatory mechanisms involved in bone destruction and different disease activity, thereby providing new insights for further exploration of RA.^[^ ^24^, ^25^, ^26^, ^27^ ^]^ Moreover, current studies have increasingly focused on investigating the underlying mechanisms of drug treatment response in RA. One study has identified differences in treatment responses to adalimumab and etanercept among RA patients using transcriptomics and DNA methylation approaches, and developed machine learning models to predict drug responses.^[^ ^28^ ^]^ In parallel, another study has reported that differential characteristics and novel biomarkers for predicting therapeutic responses in RA patients by multiomics analysis, presenting new directions for personalized therapy.^[^ ^29^ ^]^ However, there is a paucity of studies employing multiomics strategies to investigate the treatment response of csDMARDs in RA patients, and the potential associations remain largely unexplored.

Therefore, our study aimed to elucidate the differential molecular mechanisms underlying the response to csDMARDs in RA patients through a multiomics integration strategy.

## 2 Experimental Section

### 2.1 Population Recruitment and Clinical Data Collection

In this study, RA patients were recruited in the Department of Rheumatology of Dazhou Central Hospital according to the 2010 ACR‐European League Against Rheumatism (EULAR) diagnostic criteria.^[^ ^30^ ^]^ Inclusion criteria included: a. RA patients aged >18 years; b. RA patients treated with csDMARDs (mainly MTX, LEF and HCQ alone or in combination, etc.), NSAIDs (meloxicam, etoricoxib or celecoxib) and glucocorticoids. During the registration and enrolment of patients, clinical information was collected, including age, gender, disease duration, erythrocyte sedimentation rate (ESR), swollen joint count (SJC28), tender joint count (TJC28), disease activity score (DAS28), CRP, rheumatoid factor (RF), interleukin ‐ 6 (IL ‐ 6), and medication. Besides, controls were recruited from the Physical Examination Department of Dazhou Central Hospital, including: a. aged >18 years old; b. not suffering from other malignant diseases such as autoimmune diseases, blood disorders, and tumors.

### 2.2 Study Design

**Figure 1:**
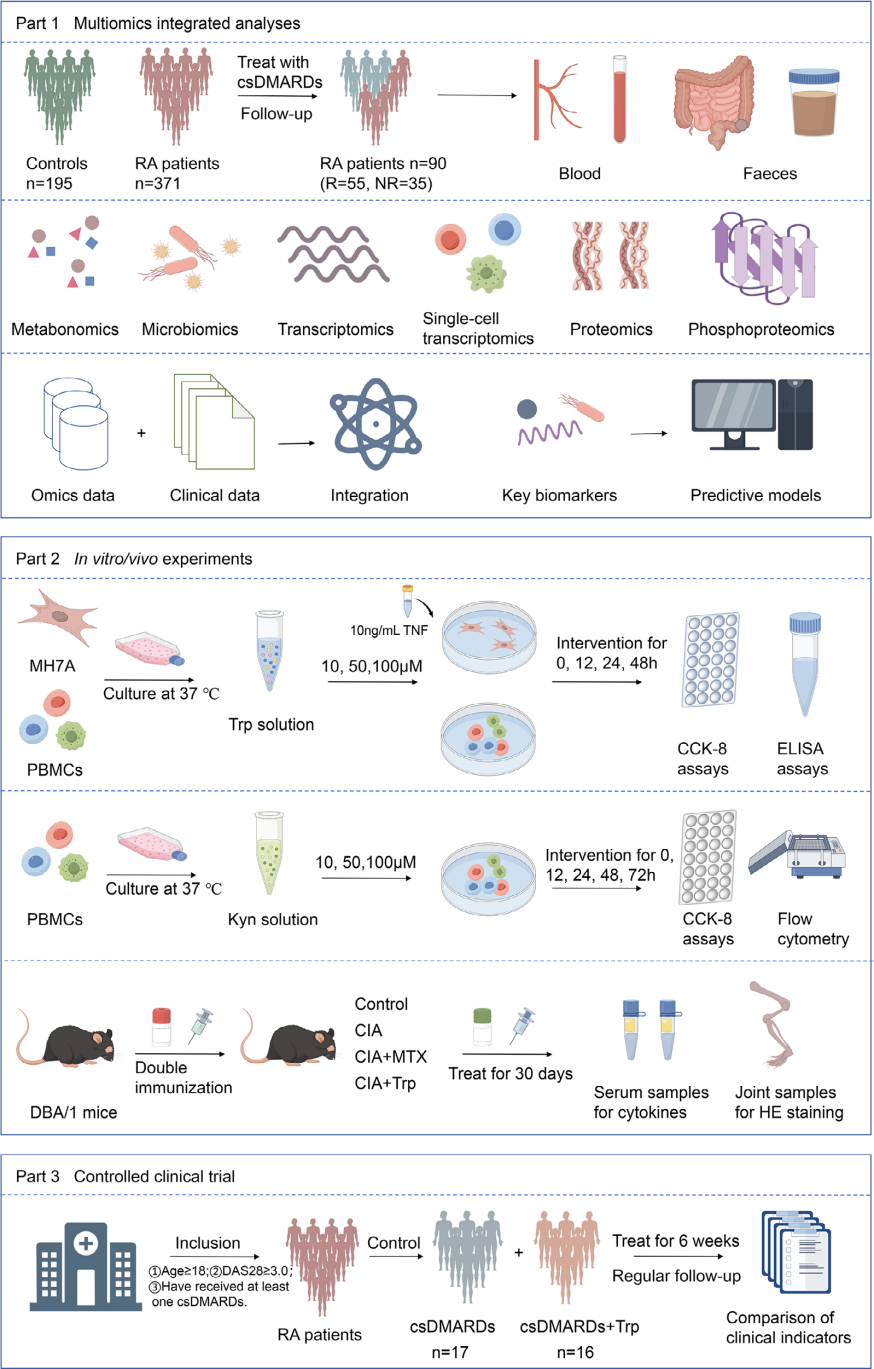
Overview of the study design. This study consisted of three parts. The first part was a multiomics integrated analyses. A total of 566 participants, including 371 RA patients and 195 controls. Follow‐up treatment data were obtained for 90 RA patients, categorized as responsers (R, n = 55) and nonresponsers (NR, n = 35). Plasma samples were utilized for metabolomics, fecal samples for microbiomics, and peripheral blood mononuclear cell samples for transcriptomics, single‐cell transcriptomics, proteomics, and phosphoproteomics assays. We conducted comparative analyses of plasma metabolic profiles and gut microbiota between RA patients and controls. Furthermore, we identified significant imbalances and deviations in plasma metabolites, gut microbiota, genes, immune cells, and proteins between responsers and nonresponsers. Our integrated analyses highlighted the critical role of Trp in influencing the therapeutic response in RA patients and we constructed predictive models for clinical response. The second part was in *vitro* */vivo* experiments validation. Based on the results of multiomics analyses, focusing on the key metabolite Trp. We assessed cell proliferation using the CCK‐8 assay and measured inflammatory cytokine expression via ELISA. Additionally, we explored the effects of its metabolite Kyn on cell proliferation and immune cells. Pathological staining and scoring of joint tissues were performed, along with cytokine expression analysis of serum samples. The third part was controlled clinical trial confirmation. A controlled clinical trial was conducted to further observe the clinical efficacy of Trp supplementation in treating RA patients. The changes in DAS28, SJC28, TJC28, ESR, CRP, and RF were compared between two groups. RA, rheumatoid arthritis; R, responsers; NR, nonresponsers; PBMCs, peripheral blood mononuclear cells; CCK‐8, cell counting kit‐8; ELISA, enzyme‐linked immunosorbent assay; CIA, collagen‐induced arthritis; MTX, methotrexate; Trp, tryptophan; csDMARDs, conventional synthesis disease modifying anti‐rheumatic drugs; DAS, disease activity score; SJC, swollen joint count; TJC, tender joint count; ESR, erythrocyte sedimentation rate; CRP, C‐reactive protein; RF, rheumatoid factor.

This study contained three main parts, and **Figure** **1** depicted an overview of the study. In part one, the multiomics analyses of clinical cohort were performed. A total of 566 participants were enrolled in this study, including 371 RA patients and 195 controls. Meanwhile, the treatment of 371 RA patients was followed up on, and DAS28 integrated three clinical parameters: SJC28, TJC28, and ESR, calculated as: DAS28 = (0.56*SQRT (TJC) +0.28*SQRT (SJC) + 0.7*LN (ESR))*1.08+0.16. The improvement of DAS28 (△DAS28) after standardized treatment over 6 months (≥24 weeks), and combined with current disease activity status to evaluate therapeutic outcomes. According to the response assessment criteria: (1) good response: △DAS28>1.2, final DAS28≤3.2. (2) moderate response: 0.6<△DAS28≤1.2, final DAS28≤5.1 or △DAS28>1.2, final DAS28>3.2. (3) no response: △DAS28≤0.6 or 0.6<△DAS28≤1.2, final DAS28>5.1. 90 RA patients met the above conditions, including 55 responsers and 35 nonresponsers. Plasma samples for metabolomics, fecal samples for microbiomics and peripheral blood mononuclear cells (PBMCs) samples for transcriptomics, single‐cell transcriptomics, proteomics, and phosphoproteomics assays were collected. Subsequently, multiomics data were integrated and predictive models of clinical response were constructed. In part two, in vitro/vivo experiments were validated. Based on the results of the multiomics analyses, the key feature Trp was screened, and experiments were used to validate its effects. HE pathological staining and scoring of joint tissues and detection of cytokines of serum samples were performed. In part three, controlled clinical trial was confirmed. Controlled clinical trial was conducted to further observe and validate the clinical efficacy of Trp‐assisted therapy for RA patients.

### 2.3 Samples Collection, Processing, and Storage

1) Blood samples from subjects were collected and after centrifugation at 3000 rpm for 10 min, the supernatant (i.e., plasma) was taken up into 1.5 mL EP tubes to obtain plasma samples, which were stored in a −80 °C refrigerator until use. 2) Blood samples from subjects were collected and transferred into 15 mL centrifuge tubes with adequate mixing, then appropriate amount of erythrocyte lysate was added, mixing and blowing with repeated inversions, and then put into a shaker for 10 min, and then centrifuged for 5 min at 3000 rpm, and then discarded the supernatant after centrifugation to get the cell precipitate, and then added 2 mL of erythrocyte lysate and mixed them adequately, and then divided into two 1.5 mL EP tubes, and then centrifuged at 3000 rpm for 5 min, the supernatant was aspirated and discarded to obtain two tubes of cells (i.e., PBMCs), one of which was mixed by blowing after addition of 0.5 mL of Trizol reagent. All samples obtained were stored directly in ‐80 °C refrigerator until use. 3) Blood samples of the subjects were obtained, then a 15 mL centrifuge tube was taken, 5 mL of blood was added, then an equal volume of PBS solution was added and mixed thoroughly to obtain the homogenate, then two 15 mL centrifuge tubes were taken and added 5 mL of lymphocyte isolate respectively, then carefully sucked up 5 mL of homogenate with a pipette and added it to the surface of isolate, and then centrifuged it for 25 min at 20 °C and 450 g/min. After centrifugation, the second layer of milky‐white lymphocytes was carefully aspirated into another 15 mL centrifuge tube with an extended 1 mL pipette tip, and 10 mL of PBS wash solution was added to the centrifuge tube to mix the cells. After three centrifugations at 20 °C and 450 g/min for 10 min to obtain the cell precipitate, 1.5 mL of cell cryopreservative solution was taken, slowly blown and mixed, then transferred to a cytofreeze tube, put into a programmed cooling box, and then putted into a −80 °C refrigerator overnight, and then on the next day, the cytofreeze tube was transferred to liquid nitrogen to be preserved until use. 4) Stool samples were collected from all subjects, dispensed into 1.5 mL EP tubes, attached to a sealing film and quickly frozen in liquid nitrogen for 5 min and then stored in a refrigerator at −80 °C until extraction.

### 2.4 Non‐Targeted LC‐MS/MS Metabolite Detection, Data Processing, and Analysis

About 100 µL of plasma sample was taken in a 1.5 mL centrifuge tube to which 400 µL of extraction solution (acetonitrile to methanol ratio 1:1) was subsequently added. After vortex mixing for 30 s, the samples were extracted by sonication at a frequency of 40 KHz for 30 min at low temperature (5 °C). Afterward, the treated samples were kept stationary at −20 °C for 30 min. Subsequently, after centrifugation of the samples at 13 000 g for 15 min at 4 °C, the supernatant was carefully aspirated and blown dry by nitrogen. Next, the samples were resolubilized using 120 µL of resolution (acetonitrile to water ratio of 1:1). The resolubilized samples were again extracted by ultrasonic extraction at 40 KHz for 5 min at low temperature (5 °C). Finally, the samples were centrifuged at 13 000 g for 5 min at 4 °C. At the end of centrifugation, the supernatant was transferred to a feed vial with an internal cannula for on‐line analysis. The samples were separated on an Agilent 1290 Infinity LC ultra‐high performance liquid chromatography (UHPLC) HILIC column, and the samples were placed in an autosampler at 4 °C throughout the analysis, while 1 QC sample was inserted every 10 samples for examining and evaluating the reproducibility and reliability of the experimental procedure. Electrospray ionization (ESI) positive and negative ion modes were used for detection. Samples were separated by UHPLC and analyzed by mass spectrometry using a TripleTOF 5600 mass spectrometer (AB SCIEX).

The raw data obtained after testing were subjected to a series of data processing steps using the professional metabolomics processing software Progenesis QI (Waters Corporation, Milford, USA), including baseline filtering, peak identification, integration, retention time correction, and peak alignment, to obtain data matrices containing retention times, mass‐to‐charge ratios, and peak intensities. The metabolite information was obtained by matching the mass spectral information with the metabolic public databases HMDB (https://www.hmdb.ca/) and Metlin (https://metlin.scripps.edu/), as well as majorbio's own library. The obtained metabolite data matrix was then uploaded to the majorbio cloud platform (https://cloud.majorbio.com) for analysis. The data were first preprocessed and the 80% rule was used to filter the variables, i.e., only those variables with more than 80% of non‐zero values in a set of samples were retained. Subsequently, the minimum values in the original matrix were used to fill in the missing values, and the response intensities of the sample mass spectral peaks were normalized using the sum normalization method to obtain a normalized data matrix. In addition, to ensure the data quality, variables with relative standard deviation (RSD) greater than 30% in the QC samples were further deleted, and the data were log10 logarithmized to finally obtain the data matrix for subsequent analysis.

The R software package ropls (Version1.6.2) was used for orthogonal partial least squares discriminant analysis (OPLS‐DA). The unit variance conversion of the OPLS‐DA data was carried out, the confidence level was 0.95, the number of replacement was 200, and the *student's t double‐tail test* was performed. According to the OPLS‐DA model, the variable weight value (VIP) was > 1, and the metabolites with p < 0.05 were identified as differential metabolites. Subsequently, metabolic pathway annotation was performed using the KEGG database (kegg_v94.2) (https://www.kegg.jp/kegg/pathway.html), pathway enrichment was performed using the topological method (Relative‐betweenness Centrality) and Python software scipy.stats analysis, and finally the pathways involved in differential metabolites were obtained.

### 2.5 DNA Extraction, Amplification, Sequencing, and Data Processing Analysis of Fecal Samples

Total microbial DNA from fecal samples was extracted according to the detailed instructions of the E.Z.N.A. soil DNA kit (Omega Bio‐tek, Norcross, GA, U.S.) and the quality and concentration of the DNA extracted was checked using 1% agarose gel electrophoresis and NanoDrop2000 equipment, respectively. The 16S V3‐V4 variable region was then amplified using a PCR instrument (ABI GeneAmp Model 9700) with amplification primers 338F (5′‐ACTCCTACGGGGAGGCAGCAG‐3′) and 806R (5′‐ GGACTACHVGGGTWTCTAAT‐3′). PCR amplification steps and reactions were as follows: first, a pre‐denaturation at 95 °C for 3 min; followed by 27 cycles, each consisting of denaturation at 95 °C for 30 s, annealing at 55 °C for 30 s, and extension at 72 °C for 45 s; completion of all cycles was followed by a stable extension at 72 °C for 10 min; and, finally, storage of the PCR products at 10 °C. 5 × TransStart FastPfu buffer 4 µL, 2.5 mM dNTPs 2 µL, upstream primer (5 uM) 0.8 µL, downstream primer (5 uM) 0.8 µL, TransStart FastPfu DNA polymerase 0.4 µL, and 10 ng of template DNA were made up to 20 µL, and in order to ensure reliability and stability, 3 replicates were performed for each sample. After completing three PCR amplifications of the same sample, all products were mixed and purified using 2% agarose gel electrophoresis back to the PCR products and the AxyPrep DNA Gel Extraction Kit (Axygen Biosciences, Union City, CA, USA). Afterward, the purified products were again quantified using 2% agarose gel electrophoresis and Quantus Fluorometer (Promega, USA). Subsequently, library construction was performed using NEXTflexTM Rapid DNA‐Seq Kit (Bioo Scientific, USA). The final library was obtained after junction linkage, magnetic bead screening to remove junction self‐linking fragments, enrichment of library template and recovery of PCR products. Finally, sequencing was performed using Illumina's Miseq PE300 platform (Shanghai Meiji Biomedical Technology Co., Ltd.). The raw data obtained from sequencing were quality controlled and spliced using fastp software (https://github.com/OpenGene/fastp, version 0.20.0) and FLASH software (http://www.cbcb.umd.edu/software/flash, version 1.2.7), respectively. The optimized sequences were obtained after quality control and stitching, and then the optimized sequences were subjected to noise reduction using the DADA2 plug‐in, and the obtained sequences were called amplicon sequence variants (ASVs). ASVs were analyzed for species taxonomy using Naive bayes classifier based on Sliva 16S rRNA database (v 138).

The preprocessed data were uploaded to the Majorbio cloud platform (https: //cloud.majorbio.com) for microbiota analysis. Firstly, the minimum number of sequences of the samples was drawn flat to obtain the data table for subsequent analysis. Subsequently, microbial community composition analysis was carried out using python‐2.7 software to screen for differential species by applying R‐3.3.1 (stat), Wilcoxon rank and two‐tailed test and bootstrap method to calculate 95% confidence intervals. Community alpha diversity was analyzed by mothur‐1.30 software, rank sum test and Tukey‐Kramer method for calculating 95% confidence intervals, and beta diversity was analyzed by using bray‐curtis distance algorithm using R software (R‐3.3.1 (vegan)), and Adonis test of intergroup differences method. Finally, the microflora were analyzed for functional prediction using PICRUSt2 (v2.2.0‐b) software.

### 2.6 Transcriptomics Sequencing and Data Processing Analysis

Total RNA was extracted from PBMCs using standard extraction methods and an Agilent 2100 Bioanalyzer was used for quality control and detection of total RNA amount and integrity. Subsequently, the library was constructed using the NEBNext Ultra RNA Library Prep Kit for Illumina. The library was amplified by PCR using fragmented mRNA templates and random oligonucleotides as primers, and the PCR products were purified using AMPure XP beads. After library construction, the libraries were initially quantified using a Qubit 2.0 Fluorometer and diluted to 1.5 ng/uL, and then tested for insert size using an Agilent 2100 bioanalyzer, and then sequenced using an Illumina NovaSeq 6000, generating 150 bp paired‐end reads. The sequencing company was NovaSeq (Beijing Nova Technology Co., Ltd.).

The raw data obtained from sequencing were quality controlled and filtered to obtain sequence data after screening out reads with junctions, containing N bases and low quality reads. Then, HISAT2 (v2.0.5) and featureCounts (1.5.0‐p3) software were used for gene alignment and gene expression quantification, respectively, to finally obtain TPM data for subsequent bioinformatic analyses. First, the data were log2 transformed and analyzed for differential genes using limma (version 3.40.6), |FC| > 2, p < 0.05 were identified as differential genes. Subsequently, differential gene annotation was performed using DAVID (https://david.ncifcrf.gov/) to obtain an annotation table. Finally, KEGG pathway enrichment analysis and GO (Gene Ontology) functional enrichment analysis were performed using KOBAS (http://bioinfo.org/kobas). In addition, immune cell infiltration analysis was performed using Cibersort software.

### 2.7 Single‐Cell Transcriptomics Sequencing and Data Processing Analysis

First, 10 uL of cell suspension was taken and mixed with Tepan blue staining solution according to 1:1 staining, put on the cell counting plate, and observed the cell number and cell viability by microscope. The cell suspension and microfluidic chip were prepared, and then the cell suspension was loaded onto the microfluidic chip, and the Singleron MatrixNEO (model SGR‐SRAf10) automated instrument was used to separate single cells into individual microtiter wells to complete the separation of individual cells, and the use of molecular labeling beads could complete the RNA capture and labeling at the same time. The GEXSCOPESingle RNA Library kit cell v2 (item No. 4 180 011) was used to reverse transcribe the mRNA captured by magnetic beads and PCR amplification to obtain a CDNA library. Then the CDNA library was segmented, splicing, PCR enrichment and fragment sorting to obtain single‐cell transcriptome library. Finally, the library was diluted to 2 nM/L and loaded onto a Novaseq6000 flow cell for double‐ended PE150 sequencing mode.

Raw sequencing data were processed using the celescope pipeline (v1.14.1) to generate gene expression profiles. The analytical workflow comprised the following key steps:

1) Data preprocessing: Reads lacking poly‐T tails in read1 were filtered using stringent quality criteria; Valid cell barcodes and unique molecular identifiers (UMIs) were extracted from qualified reads; Adapter sequences and poly‐A tails in read2 were trimmed using fastp (v1.0). 2) Sequence alignment: Cleaned reads were aligned to the GRCh38 reference genome; Gene annotation was performed using Ensembl release 99; Transcript quantification was executed through featureCounts (v1.6.2) and fastp (v2.5.3a). 3) UMI deduplication: Reads sharing identical cell barcode, UMI, and gene annotation were collapsed; UMI counts per gene per cell were quantified to construct the expression matrix. 4) Quality control: >5000 captured cells; Median genes per cell >500; Doublet rate <10%. (5) Cell filtering criteria: Cells with <200 or >2500 detected genes (nFeatures_RNA); Cells exhibiting >15% mitochondrial gene content (percent.mt); Extreme total UMI counts (nCount_RNA) outliers.

Data normalization: Following data normalization, linear dimensionality reduction was performed using principal component analysis (PCA) to generate the final processed data.

First, 13 dimensions were selected as the clustering objects and non‐linear dimensionality reduction UMAP was performed, after which FindAllMarkers was performed to find out all Cluster's marker genes and annotate the cell types and visualize the display; the R package was used to extract the information of cell types and samples in the metadata and processed to get the number and proportion of each cell type on each sample, and the proportion data was used to draw the Stacked bar graphs were drawn using the scale data; finally, monocle software was used to perform the time series analysis.

### 2.8 Proteomics and Phosphoproteomics Sequencing and Data Processing Analysis

The cell samples from the subjects were removed from the refrigerator at −80 °C, followed by the addition of an appropriate amount of DB proteolysis solution (containing 8 M urea and 100 mM TEAB, pH 8.5) with oscillatory mixing and sonication for 5 min in an ice‐water bath to fully lyse the cells, followed by centrifugation for 15 min at 4 °C and 12 000 g. After that, the supernatant was aspirated and an appropriate amount of 1 M DTT was added to the cells to be reacted for 1 h at 56 °C, and finally, a sufficient amount of IAM was added and the reaction was carried out at room temperature in a dark place for 1 h. The supernatant was collected and used according to the instructions. The supernatant was collected and the BSA standard protein solution was prepared using the Protein Quantification Kit Bradford with a concentration gradient of 0‐0.50‐0.5 µg/µL according to the instructions. About 20 µg of the sample to be tested was selected and processed by 12% SDS‐PAGE gel electrophoresis, while the protein concentration was calculated using a standard curve. About 100 µL of the sample and DB proteolysis solution were added, followed by the addition of sufficient amount of 100 mM TEAB buffer and trypsin for 4 h at 37 °C, followed by the addition of trypsin and CaCl_2_ for overnight digestion. Finally, appropriate amounts of formic acid, washing solution and eluent were added, and the filtrate was collected and lyophilized. Adding binding buffer to dissolve the lyophilized powder, after centrifugated at 12 000 g/min for 5 min at 4 °C, the supernatant was aspirated and added to the IMAC‐Fe column, incubated for 30 min at room temperature, and centrifuged for 30 s at 2000 g, then washed with washing solution and water, centrifuged and added with eluent, and the peptide eluent was collected and lyophilized. Mobile phases A (2% acetonitrile and 98% water, pH 10) and B (98% acetonitrile and 2% water, pH 10) were prepared, and the lyophilized powder was dissolved in A. Fractions were subsequently separated using an L‐3000 HPLC system with a Waters BEH C18 column (4.6 × 250 mm, 5 µm, 45 °C). The liquid chromatographic elution was then completed using a nanoElute nano‐scale UHPLC system, and the raw data generated by a tims TOF pro2 mass spectrometer with a full scanning range of m/z 100–1700, a Ramp time of 100 ms, and a Lock Duty Cycle to 100%, and PASEF settings were used to construct the DDA spectral library. Finally, the raw data for mass spectrometry were obtained using a tims TOF pro2 mass spectrometer with a full scanning range of m/z 100–1700, Ramp time 100 ms, Lock Duty Cycle to 100%, a scanning window of 25 Da, and two mobility windows.

Raw mass spectrometry data were aligned against the Homo sapiens UniProt reference proteome (release 2023_03_13) for protein identification. Data‐Independent Acquisition (DIA) spectra were processed through Spectronaut to generate spectral libraries, quantify peptide‐to‐protein matches, and perform label‐free analysis. Subsequent refinement using Spectronaut‐Pulsar applied stringent filters (99% confidence threshold for peptide‐spectrum matches; 1% FDR at peptide/protein levels) to derive high‐confidence protein expression profiles. Normalized intensity values (log2‐transformed, quantile‐adjusted) were subjected to differential analysis via the limma package, identifying proteins meeting significance thresholds (p < 0.05, |log2FC| > 1). Metascape (https://metascape.org/gp/) subsequently executed functional enrichment analysis of differentially expressed proteins, interrogating KEGG pathways and GO biological processes with significance criteria (enrichment p < 0.05).

### 2.9 Multiomics Data Integration Analysis and Predictive Models Development

In this study, the multi‐omics integration analysis was conducted using R version 3.6.3. For the metabolomics data, variables were filtered based on the criterion that non‐zero values must account for over 80% of samples. Missing values in the original matrix were imputed using the minimum value of each variable. Data normalization was performed using total sum scaling, followed by log10 transformation to stabilize variance and enhance interpretability. Microbiome data underwent quality control using FAST (version 0.20.0) and FLASH (version 1.2.7) software to obtain optimized sequences. Subsequently, noise reduction was implemented using the DADA2 plugin. Statistical analyses were conducted using Spearman correlation tests, with a significance threshold set at p < 0.05 and |r| > 0.3, ensuring robust interpretation of correlations among the integrated omics data. In addition, protein‐protein interaction network analysis using STRING (https://cn.string‐db.org/) along with visual presentation using Cytoscape software. Receiver operating characteristic curve (ROC) was used to build the prediction models and evaluated the classification performance.

### 2.10 PBMCs Isolation from RA Patients and Healthy Controls

PBMCs were isolated from fresh anticoagulated blood samples collected from RA patients and healthy controls. Whole blood was carefully layered over density gradient medium (LDS1075, Tianjin Haoyang Bio) at a 1:1 ratio and centrifuged (500 × g, 25 min, 20 °C; D3024 benchtop centrifuge, DragonLab). Following centrifugation, the mononuclear cell layer (second interface) was aspirated, transferred to a sterile tube, and washed twice with PBS (PB180327, Procell) to remove residual separation medium and platelets. Cells were resuspended in RPMI‐1640 culture medium (PM150110, Procell) supplemented with 10% FBS and 1% penicillin‐streptomycin. All procedures were performed under identical conditions for both RA and healthy donor samples to minimize technical variability.

### 2.11 Cell Culture and Maintenance

Rheumatoid arthritis synovial fibroblasts (MH7A; Ginio Biotech) and isolated PBMCs were cultured in RPMI‐1640 or DMEM (PM150210, Procell) with 10% FBS and 1% penicillin‐streptomycin. Cells were maintained at 37 °C/5% CO_2_ (Thermo incubator), passaged using trypsin‐EDTA (S310JV, Shanghai Yuan Pei), and cryopreserved in freezing medium (90% FBS + 10% DMSO; ED4540, Bomei).

### 2.12 CCK8 Viability Assay

PBMCs (1 × 10⁶ cells/mL) or MH7A cells (5 × 10⁴ cells/mL) were seeded in 96‐well plates. RA‐derived PBMCs, healthy control PBMCs, and MH7A cells were treated with TNF‐α (10 ng/mL; NeoBioscience), L‐Tryptophan (T0254‐5G, Sigma), or L‐Kynurenine (K8625‐25MG, Sigma) at 10–100 µM. After incubation (0–72 h), CCK‐8 reagent (C0038, Beyotime) diluted in serum‐free medium (1:10) was added (110 µL/well), incubated for 2 h, and absorbance measured at 450 nm (ELx800 microplate reader, BioTek). Six technical replicates per group ensured statistical robustness.

### 2.13 ELISA Sample Preparation and Detection

Supernatants from RA and healthy control PBMC cultures, as well as MH7A treatments, were collected post‐incubation, centrifuged (350 × g, 5 min; D3024 centrifuge, DragonLab), and stored at −80 °C for cytokine quantification. Sterile handling was ensured using a biosafety cabinet (OptiClean 1300, Heal Force), and equipment was sterilized with a pressure steam autoclave (DSX‐280B, Shenan Medical).

### 2.14 Flow Cytometry Assay

PBMCs from RA patients and healthy controls were stained with fluorochrome‐conjugated antibodies (BioLegend: CD3‐PE/Cy5, CD4‐PE, CD8‐FITC, CD25‐APC, FOXP3‐FITC) for 30 min at 4 °C. Cells were fixed/permeabilized (eBioscience Fixation/Permeabilization Kit: 2 511 819, 2 504 437, 2 504 548), washed, and analyzed on a Cytoflex flow cytometer (Beckman). Data were acquired using standardized gating strategies to compare immune cell subsets between groups.

### 2.15 DBA Mice Experiments

40 SPF grade male 6‐week‐old DBA/1J mice, 25±2 g, were purchased from Beijing Huafukang Bio‐technology Co. Ltd, with the production licence SCXK (Beijing) 2019‐0008. The mice were housed in the Experimental Animal Centre of Sichuan University, with suitable temperature and relative humidity, and all the mice were given free access to food and drinking water during the experiments, and the feed was provided by Beijing Huafukang Bio‐technology Co. Ltd. with a production license of Beijing Feeding Certificate (2019) 0 6076. This experimental study was approved by the Experimental Animal Ethics Committee of West China Hospital, with the ethical number of 20 220 629 002. Trp was purchased from sigma, USA, with a purity of ≥98%. MTX injection was purchased from Dazhou Central Hospital and produced by Pfizer Pharmaceutical Co.

After 40 DBA mice were acclimatized and fed for 7 days, 10 mice were randomly selected as healthy control (Control) and the remaining 30 were prepared as CIA model mice. Briefly, type II collagen (containing acetic acid at a concentration of 2 mg/ml) was slowly added dropwise to an equal volume of complete frennd's adjuvant (CFA) on ice to fully emulsify it before the experiment, and the final concentration of type II collagen was 1 mg/ml. The prepared collagen and adjuvant mixture was injected subcutaneously into the tail root of the mice, and the initial immunization on day 0 was 0.2 ml, on day 28 on the contralateral side. After 28 days of modeling, the mice were evaluated for arthritis index score, and mice with score ≥ 1 were considered to have arthritis and could be used for subsequent experiments. Mice with arthritis according to the score were randomly divided into CIA model group (CIA), MTX treatment group (CIA+MTX), and Trp treatment group (CIA+Trp), with 10 mice in each group. Together with the healthy control group there were 4 groups. The administration was started on the 28th day after the second booster immunization, MTX was used at a dose of 2 mg/kg, Trp was used at a dose of 200 mg/kg, and the healthy control group and the CIA model group were given an equal volume of saline, the route of administration was intraperitoneal injection, and the time of administration was two times per week for 30 consecutive days. At the end of the experiment, blood and joint tissue samples were collected from the mice.

### 2.16 HE Staining and Pathological Scoring of Mice Joint Tissues

Formic acid and 10% neutral formaldehyde fixative were formulated as formic acid decalcification solution in the ratio of 1:9; anhydrous ethanol was taken and diluted with purified water to form 75%, 85%, and 95% ethanol solutions, i.e., dehydration reagent; 416 mL of anhydrous ethanol and 178 mL of purified water were poured into a beaker, shaken and mixed well, and then 6 mL of concentrated hydrochloric acid was slowly added, stirred and mixed well and then formulated into a hydrochloric acid – ethanol differentiation solution. The solution was prepared as hydrochloric acid‐ethanol partitioning solution by stirring and mixing.

After the joint tissues were fixed, the joint tissues were removed and placed into the prepared formic acid decalcification solution for 1 week. After the completion of joint decalcification, the joints were processed through operations such as dehydration, embedding and sectioning, followed by xylene 5–10 min × 2 times, anhydrous ethanol 5 min × 2 times, 95% alcohol 5 min, 85% alcohol 5 min, 75% alcohol 5 min, UP water immersion for 5 min, hematoxylin staining for 10 min, rinsing in tap water for 1 min, hydrochloric acid alcohol fractionation for 5 s, rinsing in tap water for 1 min, and placing in 50 °C the blue color was returned in warm water until blue color appeared; then rinsed in tap water for 1 min, put into 85% alcohol for 3 min, stained with eosin for 3 min, washed in water for 3 s, and finally dehydrated in gradient alcohol, transparent in xylene, and sealed in neutral gum.

A Pannoramic 250 digital section scanner manufactured by 3DHISTECH (Hungary) was used for picture acquisition of the sections, and all the tissue gross lesions were first observed using low magnification, followed by selection of the area to be observed for the acquisition of 50x and 200x pictures to observe the specific lesion. Subsequently the joint tissue pictures were scored for pathological damage as indicated by the scoring criteria: (1) synoviocytes proliferation: ①no proliferation = 0, ②slight proliferation, 2–4 layers of synoviocytes = 1, ③moderate proliferation, more than 4 layers of synoviocytes = 2, ④synoviocytes over‐value, erosion of cartilage and disappearance of osteoarthritic space = 3. (2) Cellular erosion: ①no erosion = 0, ②less localized erosion = 1, ③extensive localized erosion = 2, ④extensive erosion into the joint capsule with cohesion formation = 3. (3) Vascular opacities: ①no change = 0, ②vascular opacities in two sites = 1, ③vascular opacities in four sites with erosion of the cartilage surface = 2, ④vascular opacities in more than four sites or extensive vascular opacities in two sites = 3. (4) Inflammatory cells infiltration: ①normal = 0, ②mild inflammation with 1 aggregate or less scattered leukocyte infiltration = 1, ③moderate inflammation with 2 or more leukocyte aggregates = 2, ④severe inflammation with obvious leukocyte fusion and scattered infiltration = 3. (5) Bone erosion: ①normal = 0, ②small amount of erosion, 1–2 small superficial sites = 1, ③small amount of erosion, 1–4 medium sized and deep sites = 2, ④medium erosion, 5 or more sites, localized erosion to the bone cortex = 3, ⑤severe erosion, multiple injuries, localized or complete erosion to the bone cortex = 4, ⑥extensive injuries, cortical penetration to more than 25% of the length of the bone = 5.

### 2.17 Liquid Chip Kit for IL‐1β, IL17A, IL‐6, and TNF factors

According to the procedure of the kit, first, took out the kit from the refrigerator at 4 °C, and kept it at room temperature for half an hour; then dissolved the serum samples, shook and mixed them well; then dissolved and mixed the standard, QC and serum matrix in the kit, and mixed them well, and then prepared the gradient of the standard into a fourfold dilution. Then added 200 µL Wash buffer to wash the plate, shook for 10 min, removed the liquid and shook dry, added 50 µL standard and QC to the corresponding wells, 50 µL Matrix Solution to the background wells, 25 µL Assay Buffer to the corresponding wells of the samples, 25 µL samples to the sample wells, 25 µL of magnetic beads were added to each well; finally, the plates were sealed and incubated overnight at 4 °C with shaking while avoiding light. After each addition of 200 µL of Wash Buffer, the plates were allowed to stand for 1 min and then washed three times with a magnetic plate washer, then the plates were blocked by adding 25 µL of the detection antibody to each well and incubated for 1 h at room temperature with shaking and protected from light; then the plates were blocked by adding another 25 µL of Streptavidin‐Phycoerythrin to each well and incubated for 30 min at room temperature with shaking and protected from light. After that, 200 µL of Wash Buffer was added and left for 1 min, the plate was washed three times by magnetic plate washer, 150 µL of plate reader sheath solution was added, the plate was sealed and resuspended for 5 min at room temperature by shaking and avoiding light; finally, the plate was read by MAGPIX and the standard curves of the indexes were plotted according to the instruction manual to obtain the assay data.

### 2.18 ELISA Kit for TGF‐β Factor Detection

The experimental procedure was carried out according to the procedure provided in the kit, first 1N HCl and 1.2N NaOH solution was prepared based on the instructions of the reagent, followed by removing the kit from the refrigerator at −20 °C and placing it at room temperature for 1 h. The samples to be tested were thawed, shock‐mixed, centrifuged and acidified. After diluting the samples 200‐fold by adding 100 µL of standard to the corresponding wells, 100 µL of sample dilution was added to the sample wells and incubated at 37 °C for 2.5 h with shaking. Then the liquid in the plate was removed and the plate was washed five times by adding 350 µL Wash buffer to each well, then 100 µL of the detection antibody was added to each well and incubated for 1 h at 37 °C, protected from light. Next, the plate was washed five times with 350 µL Wash buffer per well, then 100 µL HRP was added to each well and incubated at 37 °C for 45 min away from light. The plate was washed five times with 350 µL of Wash buffer per well, 100 µL of TMB was added to each well and incubated for 30 min at 37 °C under light protection, followed by the addition of 50 µL of Stop solution to each well. Finally, the assay was carried out at a wavelength of 450 nm, and the absorbance values were read within 3 min, and the standard curves were depicted according to the instructions to calculate the expression of the assayed factors.

### 2.19 Tryptophan Supplementation and Controlled Clinical Trial

To evaluate the effectiveness of Trp supplementation, RA patients were recruited according to the inclusion criteria. The inclusion criteria were as follows: ① Age between 18 and 80 years and DAS28 ≧ 3.0. ② Patients who had been treated with at least one csDMARDs (including MTX, HCQ, LEF, etc.) with poor efficacy prior to the enrolment. The exclusion criteria were as follows: ① RA patients treated with biologics. ② Patients with malignant diseases such as gastrointestinal ulcers, blood system diseases, psychiatric disorders, and tumors. ③ Patients with other autoimmune diseases such as systemic lupus erythematosus. ④ Those who had a recent history of major surgeries or traumas. The controlled clinical trial was approved by the Ethics Committee of Dazhou Central Hospital with the ethical number of 2023039 and 2024186, and all the patients participating in the study signed the informed consent form. And it has been registered in China Clinical Trial Registry with registration number ChiCTR2400081970 and ChiCTR2400094366.

RA patients were into a Trp supplementation group (i.e., Trp assisted treatment group, csDMARDs+Trp) (n = 16) and a control group (csDMARDs) (n = 17). The Trp ‐supplemented group received 2 g of Trp orally per day for 6 weeks, and the other group did not receive it orally. During the study period, RA patients continued to receive csDMARDs (MTX, orally, 10–15 mg/dose once weekly; LEF, orally, 20 mg/dose once daily; and HCQ, orally, 200–400 mg/dose once daily), while patients were allowed to use a combination of stabilized doses of glucocorticosteroids (doses ≤10 mg/day) and NSAIDs (1‐2 tablets/day). During the course of the research trial, patients were regularly followed up every 2 weeks, each time for disease assessment and checking the levels of inflammatory factors. Disease improvement was also assessed based on DAS28, SJC28, TJC28, CRP, ESR, RF, health assessment questionnaire (HAQ), and visual analogue scale (VAS) of RA patients.

### 2.20 Statistical Analysis

SPSS 25.0 software was used for *t‐test* for data that met the normal distribution in the measurement data, non‐parametric test for data that did not meet the normal distribution for comparative analysis, and chi‐square test for the count data. Data were expressed as mean ± standard deviation/standard error, with P < 0.05 being statistically significant; GraphPad prism 8.0.2 software was also used for image visualization. Additional images were drawn by Figdraw (https://www.figdraw.com/static/index.) online platform.

### Ethics Approval and Consent to Participate

This study was approved by the Medical Ethics Review Board of Dazhou Center Hospital (ID Number: 022/2021; 039/2023), and all the participants signed informed consent.

## 3 Results

### 3.1 Clinical Characteristics of the Study Population

A total of 566 participants were recruited for this study, including 371 RA patients and 195 controls. The RA patients exhibited significant abnormalities in ESR, CRP, RF, and IL‐6 levels (detailed clinical information is presented in Table S1, Supporting Information). Additionally, demographic characteristics (age and sex) were found to be similar between the 55 responsers and 35 nonresponsers. No significant differences were observed between the two groups concerning disease duration, disease activity, inflammatory markers, or medication usage (specific clinical data can be found in Table S2, Supporting Information).

### 3.2 Significant Alterations in Plasma Metabolic Profile of RA Patients and the Critical Role of Tryptophan Metabolism in the Treatment of csDMARDs

**Figure 2:**
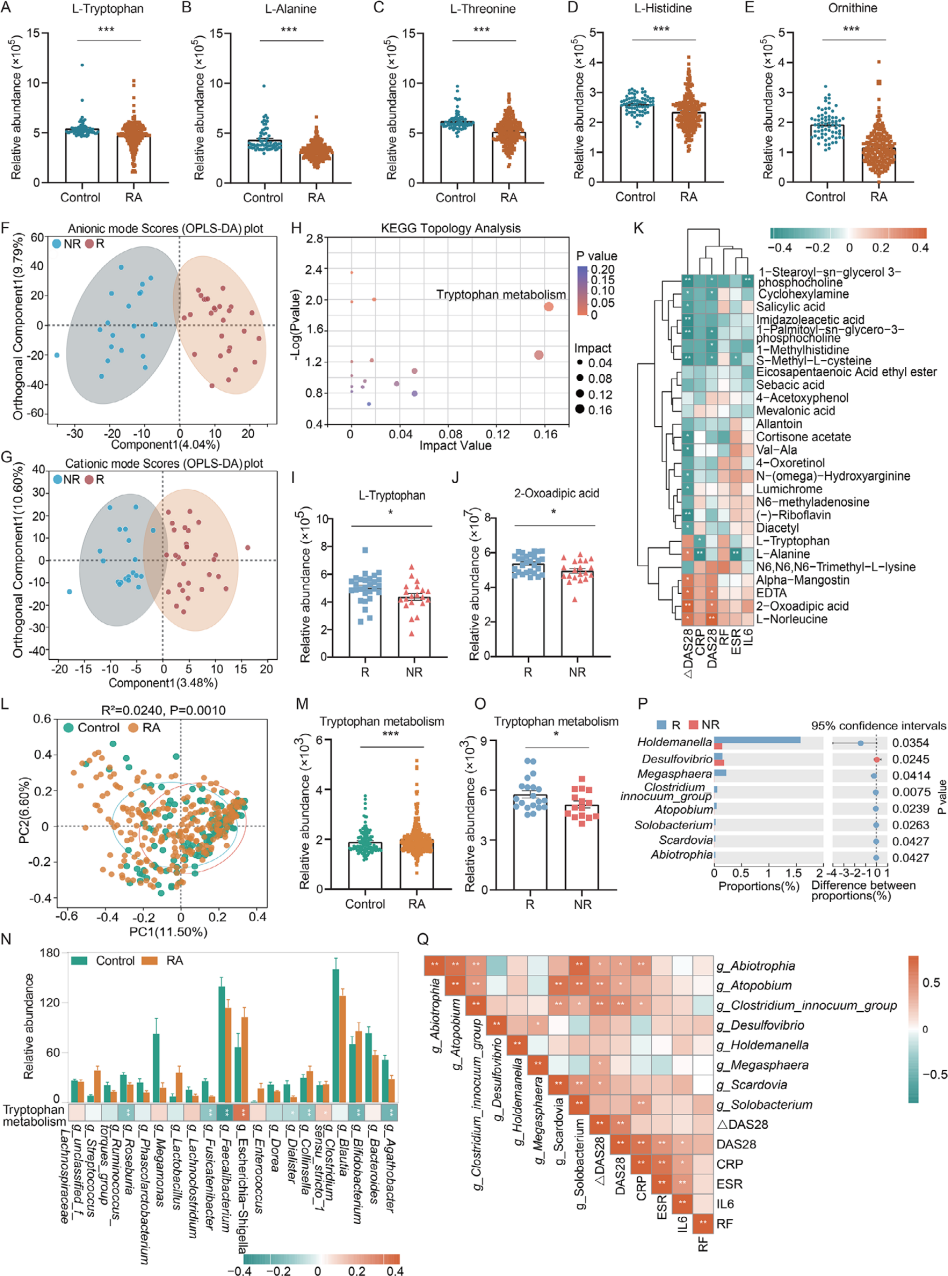
Tryptophan metabolism exhibited significant alterations in plasma metabolic profiles and gut microbiota between responsers and nonresponsers. (A–E) The relative abundance of L‐Tryptophan, L‐Alanine, L‐Threonine, L‐Histidine and Ornithine between RA and Control groups. (F,G) A clear separation of the differential metabolites in both anionic and cationic modes between response and nonresponse groups through orthogonal partial least squares discriminant analysis (OPLS‐DA), respectively. (H) KEGG pathway enrichment analysis of 27 differential metabolites between response and nonresponse groups. (I,J) The relative abundance of L‐Tryptophan and 2‐Oxoadipic acid of the tryptophan metabolism pathway. (K) The correlation analysis of 27 significant metabolites and 6 clinical characteristics. (L) The principal coordinates analysis (PCoA) demonstrated significantly distinct microbial community structures in beta diversity between RA patients and controls. (M) PICRUSt2 functional prediction analysis revealed a significant activation of the tryptophan metabolism in RA patients. (N) Relative abundance of the top 20 differential bacteria and correlation with the tryptophan metabolism between RA patients and controls. (O) Tryptophan metabolism was significantly activated in responsers through PICRUSt2 functional prediction analysis. (P) The 8 differential bacteria were identified by using Wilcox rank‐sum test between response and nonresponse groups at genus level. (Q) The correlative associations of the 8 differential bacteria with the 6 clinical indicators. (A‐K, RA, n = 244, Control, n = 69, Response, n = 28, Nonresponse, n = 20; L‐Q, RA, n = 246, Control, n = 124, Response, n = 20, Nonresponse, n = 15). RA, rheumatoid arthritis; CRP, C‐reactive protein; DAS, disease activity score; RF, rheumatoid factor; ESR, erythrocyte sedimentation rate; IL ‐ 6, Interleukin ‐ 6. *, p < 0.05; **, p < 0.01;***, p < 0.001. Data was expressed as mean ± standard error.

To investigate metabolic changes in RA patients, we analyzed plasma samples from 244 RA patients and 69 controls using metabolomics. Our analysis revealed distinct anionic and cationic patterns between the two groups (Figure S1A,B, Supporting Information) and identified 111 differential metabolites, consisting 33 upregulated and 78 downregulated metabolites (Figure S1C,D, Supporting Information). Notably, we observed significant alterations in lipid metabolites, such as glycerophosphocholine, linoleic acid, and citric acid, along with amino acid metabolites including ornithine, Trp, alanine, threonine, and histidine (Figure S1E–G, Supporting Information; **Figure** **2**A–E), suggesting the metabolic dysregulation that may contribute to RA pathogenesis.

Further exploration of treatment responses revealed distinct differences in anionic and cationic patterns between responsers and nonresponsers, correlating with notable alterations in plasma metabolites (Figure 2F,G). We identified 27 differential metabolites comprising 6 upregulated and 21 downregulated components (Figure 2H), with a significant enrichment in the Trp metabolism pathway, and the levels of both Trp and 2‐oxoadipic acid were elevated in the responser group (Figure 2I,J). Additionally, we found significant correlations between clinical indicators and various differential metabolites, including negative correlations between CRP and both Trp and alanine (Figure 2K). This indicates that fluctuations in inflammation levels may influence metabolite alterations, thereby affecting disease progression.

Overall, our findings highlight significant metabolic profile differences between responsers and nonresponsers, underscoring the critical role of Trp metabolism in the treatment of RA.

### 3.3 Dysbiosis of Gut Microbiota and Activation of Tryptophan Metabolism Pathway in RA Patients

To investigate variations in gut microbial composition and diversity among RA patients, we performed 16S rRNA sequencing of fecal samples from 246 RA patients and 124 controls. We observed significant differences in microbial community structure between the two groups (Figure 2L). However, both groups showed similar levers of community richness (Sobs index) and diversity (Shannon index) (Figure S2A,B, Supporting Information). Notably, at the phylum level, RA patients exhibited a significant reduction in the relative abundance of *p_Firmicutes* and *p_Bacteroidota*, while the abundance of *p_Proteobacteria* and *p_Verrucomicrobiota* was significantly increased (Figure S2C, Supporting Information). At the genus level, We observed decreased abundance of *g_Faecalibacterium*, *g_Bacteroides*, *g_Megamonas*, and *g_Fusicatenibacter*, alongside increases in *g_Escherichia‐Shigella*, *g_Bifidobacterium*, *g_Lactobacillus* and *g_Enterococcus* (Figure S2D,E, Supporting Information). These findings underscore significant differences in microbiota composition from phylum to genus between RA patients and controls (Figure S2F, Supporting Information). Functional predictions using PICRUSt2 indicated significant activation of pathways related to glycerophospholipid metabolism, alpha‐linolenic acid metabolism, linoleic acid metabolism, Trp metabolism, as well as the MAPK and mTOR signaling pathways in RA patients (Figure S2G, Supporting Information; Figure 2M). Furthermore, Trp metabolism showed negative correlations with *g_Bifidobacterium, g_Faecalibacterium*, and *g_Roseburia*, but positive correlations with *g_Clostridium_sensu_stricto_1* and *g_Escherichia‐Shigella* (Figure 2N).

In examining response to treatment, we found no significant differences in beta‐diversity or community richness and diversity indices between responsers and nonresponsers (Figure S2H,I, Supporting Information). However, the Trp metabolism pathway was significantly dysregulated in responsers (Figure 2O), with eight differential genera. And we also identified 8 differential genera, with *g_Holdemanella* and *g_Megasphaera* exhibiting significantly increased abundance in the response group (Figure S2J, Supporting Information; Figure 2P). In addition, a positive association was identified between *g_Holdemanella* and Trp metabolism. Clear interactions were observed among *g_Clostridium_innocuum_group*, *g_Abiotrophia*, and *g_Atopobium* with CRP, DAS28, andΔDAS28 (Figure S2K, Supporting Information; Figure 2Q), suggesting that alterations in the abundance and function of intestinal flora may influence changes in the clinical manifestations of the disease.

These data highlight significant differences in gut microbiota composition and its interactions with Trp metabolism, offering important insights for optimizing treatment strategies in RA patients.

### 3.4 Imbalances in Tryptophan Metabolism, Immune Signaling Pathways, and Immune Cells Affected the Treatment Response in RA Patients

**Figure 3:**
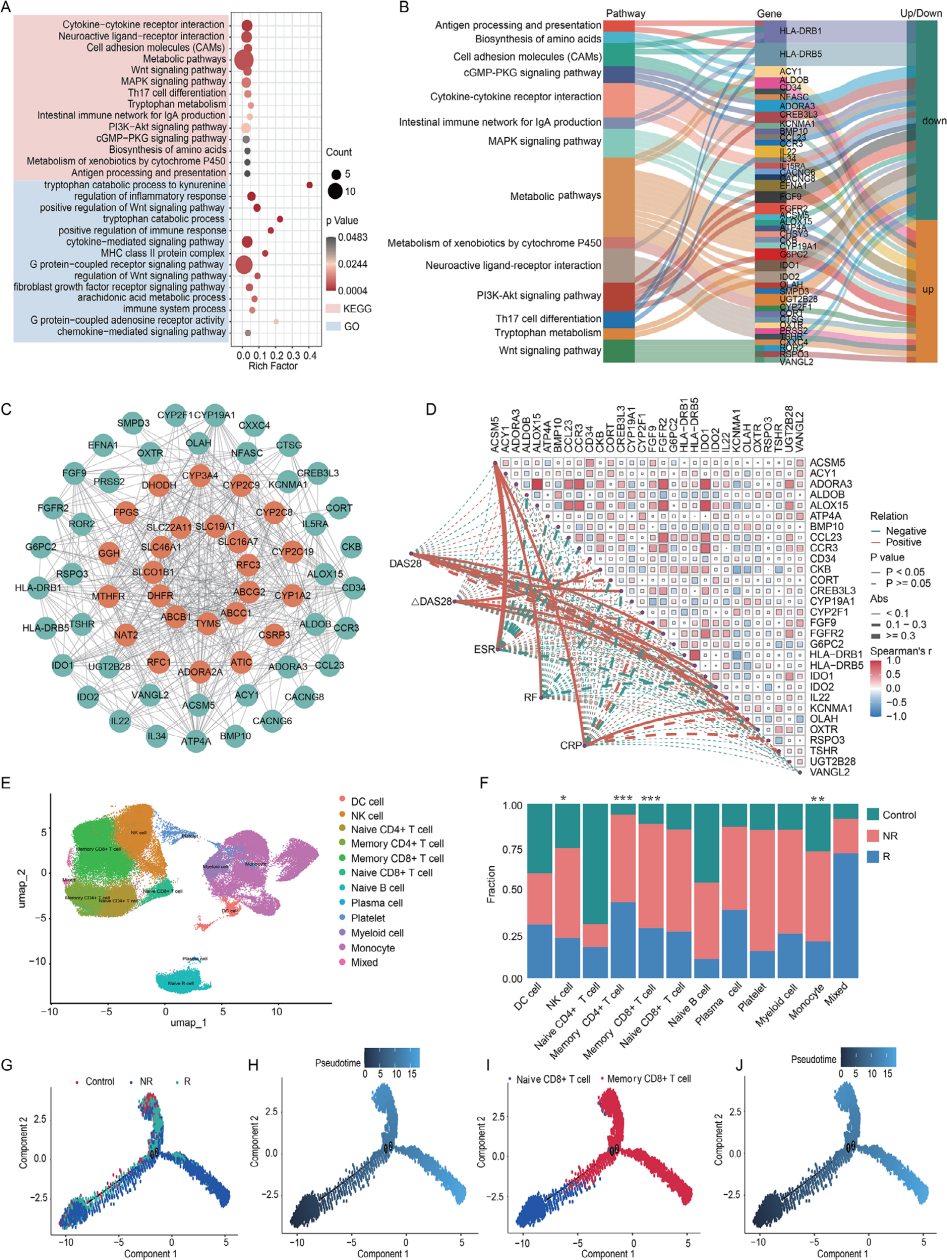
Significant dysregulation of tryptophan metabolism, immune system‐related signaling pathways and immune cells between the response and nonresponse groups. (A) 14 significantly dysregulated pathways in KEGG and GO enrichment analyses of 196 differentially expressed genes between response and nonresponse groups, separately. (B) Sankey diagram exhibited 42 differentially expressed genes of 14 KEGG pathways. (C) Protein‐protein interaction network of the association between 42 differentially expressed genes and 25 treat‐related genes. (D) The correlation heatmap illustrated the interaction of 30 differentially expressed genes with 5 clinical indicators. (E) Uniform manifold approximation and projection (UMAP) analysis displayed a clear distinction of 12 cell types based on single‐cell transcriptomics data. (F) The expression of 12 cell types between control, response and nonresponse groups. (G,H) The dynamics and migratory trajectories of immune cells between the control, response and nonresponse groups. (I,J) The dynamics and migratory trajectories of memory CD4+ T cells and memory CD8+ T cells immune cells. (A‐D, R, Response, n = 24, NR, Nonresponse, n = 11; E‐H, Response, n = 3, Nonresponse, n = 4, control, n = 2). **, p < 0.01;***, p < 0.001. Data was expressed as mean±standard error.

To investigate gene expression differences between the response and nonresponse groups, we performed transcriptomic sequencing on PBMCs from 35 RA patients. We identified 196 differentially expressed genes, including 89 upregulated and 107 downregulated (Figure S3A, Supporting Information). KEGG enrichment analysis demonstrated significant dysregulation in metabolic pathways and immune system‐related signaling pathways, notably, Trp metabolism, the Wnt signaling pathway, the MAPK signaling pathway, the PI3K‐Akt signaling pathway, and cytokine‐receptor interactions. GO enrichment analysis revealed alterations in processes pertinent to Trp metabolism, inflammatory response regulation, Wnt signaling pathways, cytokine‐mediated signaling pathways, G protein‐coupled receptor signaling pathways, and immune system processes (**Figure** **3**A). These findings indicated that variations in Trp metabolism and immune system functionality may critically influence treatment outcomes. Among the differentially expressed genes, 42 showed notable links to treatment response, comprising 22 downregulated and 20 upregulated genes, with significant interactions evident among them (Figure 3B,C; Figure S3B, Supporting Information). Furthermore, positive correlations were found between acyl‐CoA synthetase medium chain family member 5 (ACSM5) and CRP, ESR, and RF. Similarly, correlations emerged between DAS28 and several differentially expressed genes, including cAMP responsive element binding protein 3 like 3 (CREB3L3), fibroblast growth factor receptor 2 (FGFR2), IDO2, and oxytocin receptor (OXTR) (Figure 3D), underscoring the impact of inflammation on gene expression changes. Given the close relationship between the development of RA and the functional status of the immune system regulated by immune cells, we identified significant differences in CD8 T cells, M0 macrophages, and mast cells (Figure S3C,D, Supporting Information).

We focused on the immune cell status profile across control, response, and nonresponse patients. PBMCs samples were collected from nine participants for single‐cell transcriptomics sequencing. After data filtering and quality control, we ultimately obtained a total of 75773 immune cells, which were annotated for cell type and identified 12 cell clusters, including DC cell, NK cell, naive CD4+ T cell, memory CD4+ T cell, naive CD8+ T cell, memory CD8+ T cell, naive B cell, plasma cell, platelet, myeloid cell, and monocyte, and other immune cell types (Figure S4A–D, Supporting Information; Figure 3E). Noteworthy differences in the frequencies of memory CD4+ T cells, memory CD8+ T cells, monocytes, and NK cells were observed between responsers and non‐responsers (Figure 3F), suggesting that immune cell dynamics may substantially influence treatment efficacy. Additionally, we noted alterations in developmental trajectories, particularly for naive and memory CD8+ T cells (Figure 3G–J), indicating that immune dysregulation can significantly affect therapeutic responses. We employed SCENIC analysis to investigate key transcription factors within the gene regulatory network (GRN) and selected differential genes related to Trp metabolism in CD4+ T cells, CD8+ T cells, and monocytes as target genes. Through cell subsampling and ROC testing, we identified four transcription factors in CD4+ T cells (CNOT3, HMGB1, JUNB, RAD21), two in CD8+ T cells (BATF, HMGA1), and five in monocytes (CEBPA, CEBPB, CEBPD, MAFB, SPI1) (Figure S5A,B, Supporting Information). Subsequent KEGG enrichment analysis revealed that the transcription factors in CD4+ T cells primarily regulated the TNF and MAPK signaling pathways, while those in CD8+ T cells were significantly enriched in the Rap1 signaling pathway. In monocytes, the transcription factors were notably enriched in immune signaling pathways, including the NF‐kappa B, TNF, and Toll‐like receptor signaling pathways (Figure S5C–E, Supporting Information). These findings highlight the critical regulatory roles of HMGB1, JUNB, BATF, HMGA1, CEBPB, CEBPD, MAFB, and SPI1 in the activation of immune cells and immune pathways.

These results revealed that dysregulation in Trp metabolism, immune signaling pathways, and immune cell profiles contributed to an imbalance in the immune system, which was crucial for regulating RA treatment outcomes.

### 3.5 Dysregulation of Immune System‐Related Signaling Pathways Between Response and Nonresponse Groups

**Figure 4:**
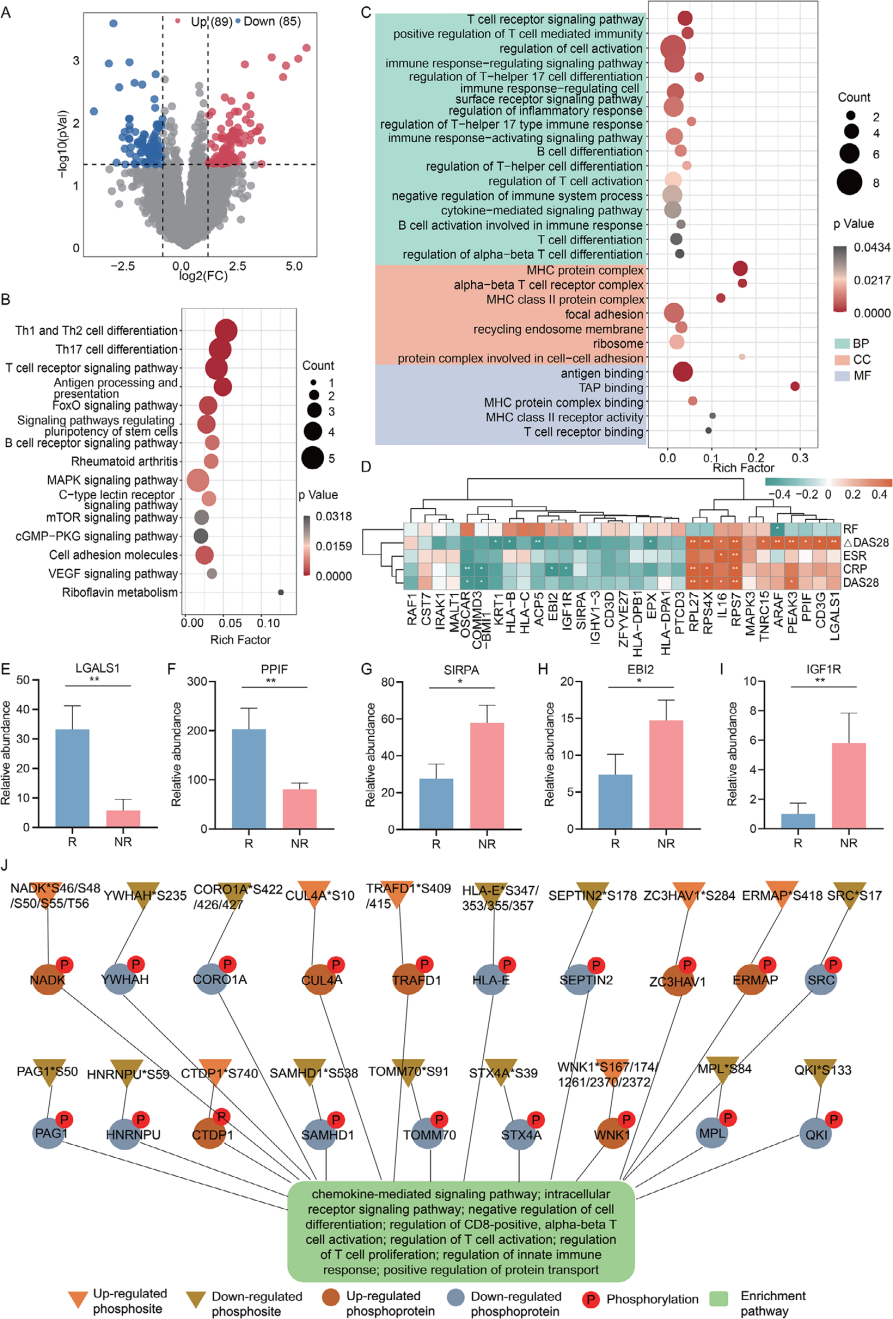
The regulatory imbalance of immune system and immune‐related signaling pathway between response and nonresponse groups of RA patients. (A) The volcano plot showed the 174 significant proteins with 89 upregulated and 85 downregulated proteins. (C) The KEGG and GO enrichment analysis of 174 differential expressed proteins. (D) The correlation analysis of 31 significant proteins and 5 clinical characteristics. (E‐I) The expressions of LGALS1, PPIF, SIRPA, EBI2, and IGF1R between the groups. (J) Relational network map demonstrated the connection of differentially phosphorylated proteins with differentially phosphorylated sites. (A‐I, R, Response, n = 12, NR, Nonresponse, n = 9). *, p < 0.05; **, p < 0.01. Data was expressed as mean±standard error.

To further explore the changes in protein and phosphorylation levels between responsers and nonresponsers, this study conducted proteomic analysis on samples from 21 RA patients. A total of 174 differentially expressed proteins were identified, including 89 upregulated and 85 downregulated proteins (**Figure** **4**A). KEGG analysis revealed significant dysregulation of pathways related to immune cell activation, T/B cell receptor signaling, and other immune‐related signaling pathways (Figure 4B,C). Additionally, 31 significantly dysregulated differential proteins showed a notable correlation with clinical indicators. Specifically, △DAS28 exhibited positive correlations with LGALS1 and PPIF, while it correlated negatively with SIRPA. EBI2 and IGF1R also demonstrated significant negative correlations with CRP (Figure 4D). Compared to the nonresponser group, the expression levels of LGALS1 and PPIF were significantly increased in the responser group, whereas SIRPA, EBI2, and IGF1R showed significant decreases (Figure 4E‐I). These findings suggested that variations in differential protein levels may interfere with treatment responses in RA patients.

Given the significant alterations in protein expression levels, we further investigated phosphorylation modifications by collecting samples from three RA responsers before and after treatment for phosphoproteomic analysis. We identified 147 differentially phosphorylated proteins, including 64 upregulated and 83 downregulated proteins, which predominantly enriched pathways regulating T cell differentiation, proliferation, and activation, critically impacting RA therapies (Figure S6A,B, Supporting Information). Specifically, compared to pre‐treatment levels, the expressions of p‐CORO1A, p‐HLA‐E, p‐HMGB1, p‐SRC, p‐PAG1, p‐SAMHD1, and p‐YWHAH significantly decreased post‐treatment, while p‐TRAFD1, p‐WNK1, and p‐FCHO1 saw marked increases. These protein level changes indicated that dysregulation of immune signaling pathways could disrupt cell cycle processes and apoptotic regulation, thereby affecting drug efficacy in RA patients (Figure S6D–M, Supporting Information).

Moreover, we identified 553 differential phosphorylation sites between pre‐ and post‐treatment, subsequently selecting 19 key phosphorylated proteins corresponding to 33 differential phosphorylation sites. These critical proteins play vital roles in immunological signaling pathways and in biological processes such as T cell proliferation, activation, and differentiation, thus regulating immune system functions (Figure S6C, Supporting Information; Figure 4J).

The results elucidated that the significant changes in protein expression and phosphorylation in RA patients, indicating their essential roles in modulating cellular and immune signaling pathways, ultimately influencing treatment responses.

### 3.6 Molecular Network Modulation of RA Treatment Response Map Based on Multiomics Data and Development of Clinical Prediction Models

**Figure 5:**
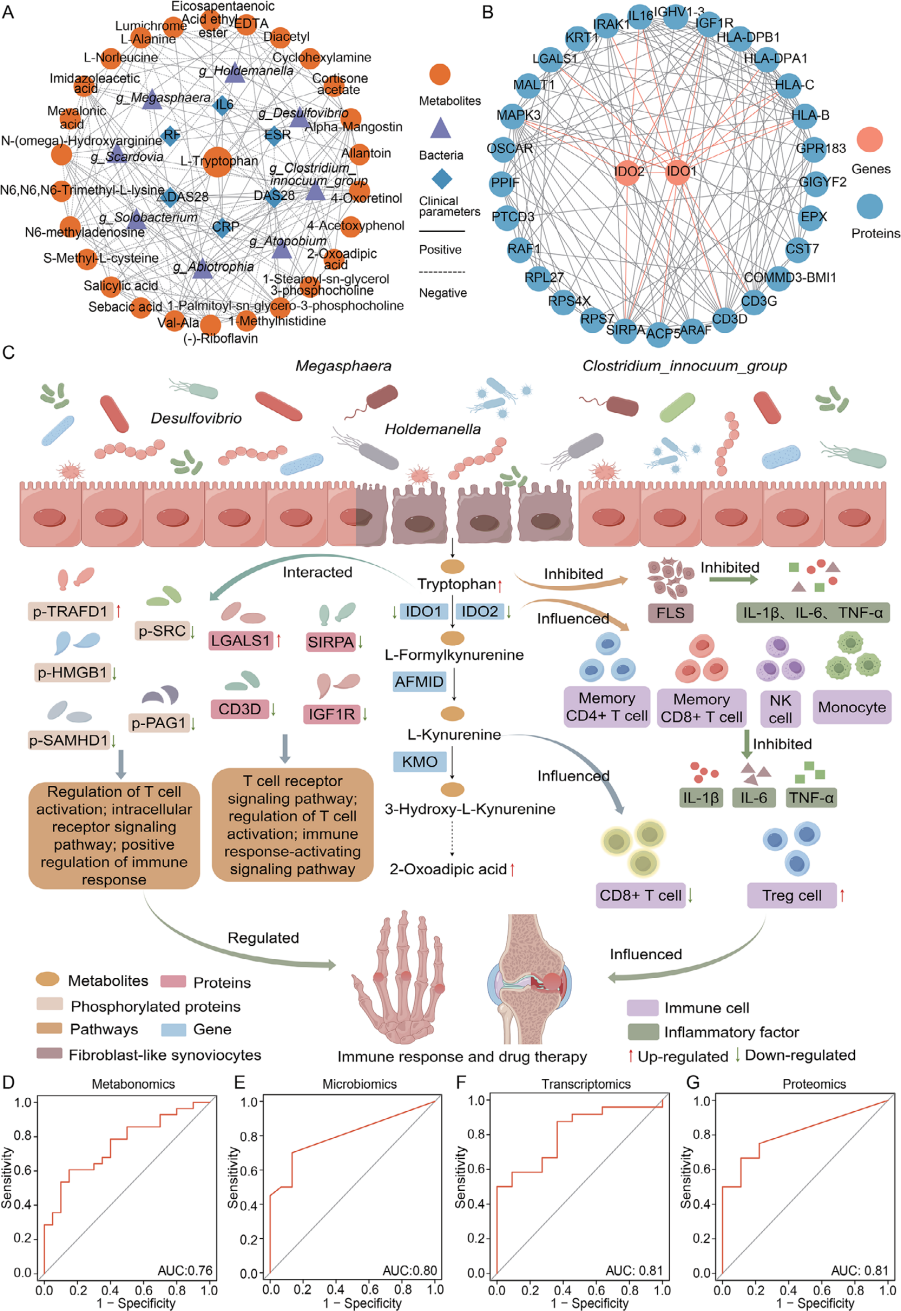
Tryptophan metabolism influenced csDMARD therapy in RA patients through multiomics analyses. (A) The correlation network showed the interactions between 27 differential metabolites, 8 differential bacteria, and 6 clinical indicators. (B) Protein–protein interaction network diagram of key genes IDO1, IDO2 and 31 key proteins. (C) The schematic map depicted the links of the tryptophan metabolism pathway, the gut microbiota, genes, proteins, phosphorylated proteins, and immune cells that collectively influence drug therapy response to RA. (D–G) The receiver operating characteristic (ROC) curve demonstrated the AUCs of metabolomics, microbiomics, transcriptomics, and proteomics models to differentiate between response and nonresponse patients, respectively.

To prove the potential molecular mechanisms underlying treatment response, we performed Spearman correlation analyses on differential metabolites, significant intestinal flora, and clinical indicators between response and nonresponse groups. We found that both positive and negative correlations among metabolites, gut microbiota, and clinical indicators, indicating their potential interplay in influencing treatment responses in RA patients (**Figure** **5**A). Furthermore, we identified that the differential genes IDO1 and IDO2 exhibited robust interactions with differential proteins, immune‐related molecules, and phosphorylated proteins, highlighting a close relationship among these variations (Figure 5B; Figure S7A,B, Supporting Information). The multi‐omics analysis centered on the Trp metabolism pathway, revealing its intricate regulatory interactions with gut microbiota, genes, and immune cells, which may impact immune responses and therapeutic outcomes. On one hand, key genes in the Trp metabolism pathway, IDO1 and IDO2 interact with marker genes of memory CD4+/CD8+ T cell, NK cell, and monocyte, modulating immune cell functions and subsequently affecting immune response and treatment changes. Additionally, Trp is known to inhibit the proliferation of fibroblast‐like synoviocytes and the secretion of inflammatory factors. On the other hand, the interactions of IDO1 and IDO2 with differential and phosphorylated proteins participate in regulating T cell activation and the dysregulation of immune signaling pathways (Figure 5C). These interconnections among the molecules likely play a significant role in modulating immune responses and drug treatment efficacy.

To predict treatment response in RA patients, we identified that Trp and the Trp metabolism pathway play crucial roles in therapeutic outcomes. Accordingly, we established multi‐omics clinical prediction models. In the metabolomics model, Trp and 2‐oxoadipic acid from the Trp metabolism pathway were selected as biomarkers, yielding an AUC of 0.76, with sensitivity and specificity values of 0.85 and 0.64, respectively (Figure 5D). The microbiome model selected the genera *g_Clostridium_innocuum_group* and *g_Holdemanella*, which were correlated with Trp and its metabolism, achieving an AUC of 0.80 with sensitivity and specificity values of 0.93 and 0.70, respectively (Figure 5E). In the transcriptomics model, the key enzyme IDO1 from the Trp metabolism pathway was chosen, resulting in an AUC of 0.81, with sensitivity and specificity at 0.91 and 0.75, respectively (Figure 5F). For the proteomics model, LGALS1, a protein that interacts strongly with IDO1, was selected, also achieving an AUC of 0.81, with sensitivity and specificity of 1.00 and 0.67, respectively (Figure 5G). These results indicated that all models demonstrated good predictive capabilities and held diagnostic value for distinguishing between responsers and nonresponsers.

The integrated analysis of multi‐omics data revealed a molecular network landscape whereby Trp and its metabolism pathway may influence and regulate treatment responses in RA patients. The clinical prediction models exhibited strong performance in differentiating the two patient groups, offering significant diagnostic potential.

### 3.7 Tryptophan Significantly Inhibited Cell Proliferation and Secretion of Inflammatory Factors

**Figure 6:**
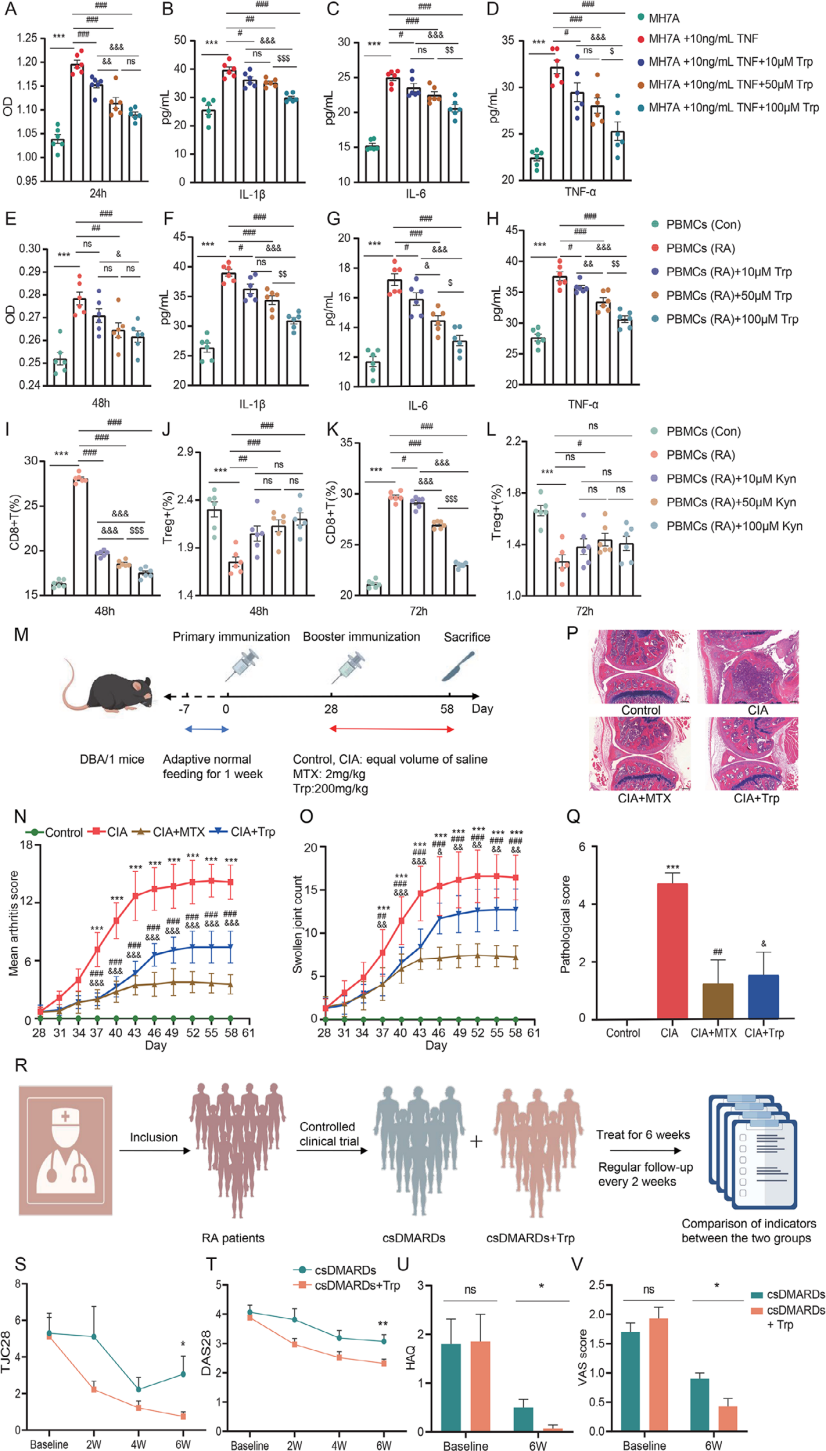
Tryptophan significantly inhibited inflammation and played an anti‐inflammatory role in cells, mice and clinical populations. (A) The proliferative capacity of MH7A synovial cells after 24 h of intervention with varying concentrations of Trp. (B–D) The expression levels of IL‐1β, IL‐6, and TNF‐α in MH7A synovial cells after 24 h of intervention with varying concentrations of Trp. (E) The proliferative capacity of PBMCs after 48 h of intervention with varying concentrations of Trp. (F–H) The expression levels of IL‐1β, IL‐6, and TNF‐α in PBMCs after 24 h of intervention with varying concentrations of Trp. (I,J) The proportions of CD8+ T cells and Treg cells in PBMCs after 48 h of intervention with varying concentrations of Kyn. (K‐L) The proportions of CD8+ T cells and Treg cells in PBMCs after 72 h of intervention with varying concentrations of Kyn. (M) Illustration of the in vivo experiment. (N,O) Arthritis score and swollen joint counts in mice. Control = 10, CIA = 7, CIA+MTX = 9, CIA+Trp = 10, data was expressed as mean±standard deviation. (P‐Q) HE pathological staining and scores of mice knee joint tissues. Control = 8, CIA = 7, CIA+MTX = 8, CIA+Trp = 9, data was expressed as mean±standard error. Scale bar was 50 µm. * represented control group versus CIA group; ***, p < 0.001. # represented MTX group versus CIA group; *#, p < 0.05; ##, p < 0.01; ###, p < 0.001*. & represented Trp group versus CIA group. *&, p < 0.05; &&, p < 0.01; &&&, p < 0.001*. (R) The flowchart of controlled clinical trial. (S,T) Changes in DAS28 and TJC28 from baseline to week 6 between the csDMARDs + Trp group and the csDMARDs group. (U‐V) The apperent improvement of HAQ and VAS scores in csDMARDs+Trp after 6 weeks. csDMARDs = 17, csDMARDs+Trp = 16. *, p < 0.05; ns, not significant. Data was expressed as mean±standard error. PBMCs, peripheral blood mononuclear cells; CIA, collagen‐induced arthritis; MTX, methotrexate; Trp, tryptophan; RA, rheumatoid arthritis; csDMARDs, conventional synthesis disease modifying anti‐rheumatic drugs; DAS, disease activity score; TJC, tender joint count; HAQ, health assessment questionnaire; VAS, visual analogue scale.

To further elucidate the functions and effects of Trp, we intervened with Trp in MH7A synovial cells and PBMCs. The results revealed a time‐ and dose‐dependent trend in the action of Trp on both cell types. After 24 h of intervention, Trp significantly inhibited the proliferation of MH7A synovial cells and markedly reduced the expression levels of IL‐1β, IL‐6, and TNF‐α (Figure S8A–C, Supporting Information; **Figure** **6**A–D). Similarly, after 48 h of treatment with Trp in PBMCs, we observed a time‐ and dose‐dependent inhibition of PBMC proliferation, along with significant decreases in IL‐1β, IL‐6, and TNF‐α levels (Figure S8D–F, Supporting Information; Figure 6E–H).

Furthermore, to explore the role of the Trp metabolic pathway, we treated PBMCs with the Trp metabolite kynurenine (Kyn). Our findings indicated that after 48 and 72 h of intervention, PBMCs proliferation was significantly diminished, exhibiting a time‐ and dose‐dependent relationship. Flow cytometry analysis revealed that both after 48 and 72 h of Kyn treatment, the proportion of CD8+ T cells significantly decreased, while the percentage of Tregs increased markedly (Figure S8G–K, Supporting Information, Figure 6I–L).

These results highlighted the critical regulatory roles of Trp and Kyn in modulating cell proliferation, inflammatory cytokine secretion, and changes in immune cell populations, thereby influencing immune responses and therapeutic outcomes.

### 3.8 Supplementation with Tryptophan Alleviated Joint Inflammation in CIA Mice and RA Patients

In light of findings from multiomics analyses that underscored the essential role of Trp in the treatment of RA, we conducted in vivo experiments to evaluate the effects of Trp (Figure 6M). Our results indicated that both the Trp‐treated group and the MTX‐treated group had significantly lower arthritis scores and reduced swollen joint counts compared to the model group (Figure 6N,O). Subsequently, histological examination revealed that mice in the model group exhibited compromised articular cartilage surfaces, increased chondrocyte numbers, areas of partial necrosis in the bone trabeculae, elevated osteoclast presence, and notable infiltration of inflammatory cells with vascular congestion. In contrast, the Trp‐treated group showed only partial cartilage compromise and minimal inflammatory cell infiltration in necrotic areas (Figure 6P). Moreover, pathological scores for the joints in both the MTX‐treated and Trp‐treated groups were significantly lower than those in the model group (Figure 6Q). We also found that cytokine levels were notably altered in the model group compared to controls, with increased expression of IL‐1β, IL‐17A, IL‐6, and TNF. The MTX‐treated group demonstrated significant reductions in IL‐1β and IL‐6 levels, while the Trp‐treated group showed a marked decrease in TGF‐β expression. Although a trend toward reduced levels of IL‐1β, IL‐17A, IL‐6, and TNF was evident in the Trp‐treated group, these changes did not reach statistical significance (Figure S9A–E, Supporting Information).

In this study, we identified that the levels of Trp in responsers were significantly higher than those in nonresponsers based on multiomics analyses. Moreover, in vitro experiments showed that Trp significantly inhibited cell proliferation and the secretion of inflammatory factors, and in vivo experiments demonstrated that Trp treatment significantly reduced arthritis scores and effectively improved the inflammatory state. Collectively, these findings underscored the critical role of Trp in modulating the response to drug therapy. Consequently, we conducted a preliminary controlled clinical trial involving RA patients who received 2 g of oral Trp daily for 6 weeks, with follow‐up visits every 2 weeks to evaluate disease status and measure ESR, CRP, and RF levels (Figure 6R). As anticipated, Trp supplementation significantly improved clinical symptoms in RA patients. Notably, participants receiving Trp‐assisted therapy exhibited significantly lower DAS28 and TJC28 compared to those in the csDMARDs group (Figure 6S,T). We also observed improvements in serum inflammatory markers, including ESR, CRP, and RF in RA patients, although these changes did not reach statistical significance (Figure S10A–D, Supporting Information). Notably, there were no significant differences in the expression levels of ALT, AST, BUN, or Scr between the two groups (Figure S10E–H, Supporting Information). Additionally, both HAQ and VAS scores were significantly lower in RA patients receiving Trp supplementation than in those who did not (Figure 6U,V).

Our findings indicated that Trp supplementation effectively improved disease activity and inflammatory status in RA, suggesting its potential as an anti‐inflammatory therapeutic agent.

## 4 Discussion

Our findings identified significant alterations in the metabolic profiles between the response and nonresponse groups, with differentially regulated metabolites enriched in the Trp metabolism pathway. We observed that Trp metabolism was significantly activated in the response group, and differential bacteria exhibited substantial correlations with both Trp metabolism and clinical indicators. Furthermore, we identified Trp metabolism and immune system‐related signaling pathways were dysregulated, alongside an imbalance of immune cell populations between the response and nonresponse groups. Importantly, our multiomics molecular map demonstrated a potential association between Trp and its metabolic pathway in regulating treatment response in RA patients. Clinical models for predicting response showed good predictive ability based on multiomics data. In vitro experiments showed that Trp significantly inhibited the proliferation of MH7A synovial cells and PBMCs, while notably reducing the levels of inflammatory factors IL‐1β, IL‐6, and TNF‐α. Moreover, we confirmed that Trp treatment significantly reduced disease activity scores and improved joint inflammatory status in both CIA mice and RA patients through in vivo experiments and controlled clinical trial.

An increasing number of studies have highlighted significant alterations in Trp metabolism across various diseases, including RA, dysregulation of Trp metabolism plays a crucial role in the pathogenesis and therapeutic exploration of RA.^[^ ^31^, ^32^, ^33^ ^]^ In this study, we observed a significant dysregulation in plasma metabolic profiles between responsers and nonresponsers, with responsers exhibiting higher levels of Trp, and Trp metabolic pathway was significantly disrupted between them. Similarly, a study found that metabolic profiling of tocilizumab treatment showed higher Trp level in responsers than in nonresponsers, aligning with our findings and suggesting that variations in Trp level may influence the therapeutic response in RA.^[^ ^34^ ^]^ Experimental studies have confirmed a close relationship between disease progression and amino acid metabolism disorders in RA, indicating significantly reduced Trp concentrations in rats.^[^ ^35^ ^]^ We also identified changes in amino acid levels between responsers and nonresponsers, underscoring the impact of these fluctuations on disease status and their essential role in immune and inflammatory responses. Furthermore, our in vivo experiments demonstrated that Trp supplementation significantly reduced inflammation scores in mice, alleviating arthritic symptoms. Previous research has indicated decreased Trp levels in RA patients, with metabolic changes correlating with disease activity, inflammation levels, RF, and potentially predicting MRI scores for assessing joint pathology.^[^ ^16^, ^18^, ^36^ ^]^ These findings indicate that variations in Trp levels are closely associated with inflammatory state and prognosis of RA.

In this study, we demonstrated that Trp and its metabolic pathway played a critical role in the therapeutic response of RA patients to csDMARDs. As an essential amino acid, Trp is vital for various physiological functions and regulatory mechanisms within the human body.^[^ ^10^ ^]^ Trp metabolism primarily occurs via the kynurenine pathway, mediated by enzymes such as IDO1 and IDO2, which are closely linked to immune regulation, neuronal function, and gut homeostasis.^[^ ^14^ ^]^ These key rate‐limiting enzymes not only participate in kynurenine metabolism but also influence the activation and differentiation of regulatory T cells, thereby modulating immune responses.^[^ ^13^ ^]^ In autoimmune diseases, IDO1‐mediated Trp catabolism acts as a regulatory mechanism to counteract excessive immune activation, promoting anti‐inflammatory programs in plasmacytoid dendritic cells. IDO2 can also be induced in B cells and antigen‐presenting cells, potentially improving disease conditions by inhibiting T and B cell activity, making it a promising therapeutic target.^[^ ^14^, ^37^, ^38^ ^]^ Our findings of significantly reduced expression levels of IDO1 and IDO2 in responders suggest that alterations in Trp metabolism may impact therapeutic outcomes, underscoring Trp's crucial role in regulating inflammatory responses and immune reactions

At present, integrative multiomics approaches provide valuable insights into the pathogenesis and therapeutic responses in RA. A study has demonstrated the immune cell characteristics in autoimmune diseases (RA, systemic lupus erythematosus, and Sjögren's syndrome) with employing transcriptomics, single‐cell transcriptomics, and experimental methods, indicating that changes of immune cells influence the onset and progression of diseases.^[^ ^39^ ^]^ Additionally, longitudinal monitoring of drug responses at the multiomics level demonstrated that treatment affects molecular levels across transcriptomics, serum proteomics, and immunophenotypes in RA patients.^[^ ^22^ ^]^ Our research identified differential changes between responsers and nonresponsers, suggesting that Trp metabolism significantly impacts RA treatment. Omics features play a crucial role in predicting therapeutic responses, with previous studies indicating that metabolites and gut microbiota can serve as effective biomarkers.^[^ ^40^, ^41^, ^42^ ^]^ Moreover, our predictive models demonstrated comparable capabilities, underscoring the importance of integrative multi‐omics techniques in elucidating RA pathogenesis and treatment responses, thereby supporting enhanced clinical management.

Nevertheless, this study had some limitations. First, the small size of responsers and nonresponsers necessitates larger populations in future research for more comprehensive analysis and validation. Second, while our study demonstrated that Trp and its metabolism played a key role in the treatment of RA patients at both phenotypic and genetic levels, the specific molecular mechanisms require further investigation. Third, the performance and predictive capability of multiomics clinical models need to be externally validated to assess their accuracy and stability. Finally, the limited number of clinical samples and follow‐up duration in the trial, along with the variability in duration and swollen joint counts between the two groups, highlights the need for larger sample sizes and longer follow‐up periods in future research and exploration.

## 5 Conclusion

In summary, this study demonstrated significant dysregulation at the phenotypic, molecular, and immune cell levels between the response and nonresponse groups through multiomics analyses. These findings underscore the critical regulatory role of Trp in mediating treatment response in RA. Furthermore, we confirmed its role in improving inflammation in vivo, in vitro and in population trials, indicating its potential as a therapeutic strategy and providing new insights for modifying and optimizing clinical treatment protocols.

## Conflict of Interest

The authors declare no conflict of interest.

## Author Contributions

C.J., J.Z., J.W., and Y.Z. contributed equally to this work. F.Z. and C.J. conceived the study. C.J. performed data analysis and wrote the manuscript. J.Z., J.W., and Y.Z. recruited patients. J.C., H.W., H.L., K.X., J.H., X.Z., Y.W., S.L., T.W., X.H., Q.Z., Y.Z., T.Z., M.T., Q.Y., and J.Z. collected data and information. J.Z. and J.S. instructed the experiments. X.D., Y.L., B.L., X.Z., and Q.W. provided single‐cell technical support and data analysis. F.Z., J.Z., and J.W. supervised the study. All authors approved the final version for submission.

## Supporting information



Supporting Information

Supporting Information

Supporting Information

Supporting Information

## Data Availability

The data that support the findings of this study are available from the corresponding author upon reasonable request.
