# Supplementary material for: Multiomics Analyses Reveal an Essential Role of Tryptophan in Treatment of csDMARDs in Rheumatoid Arthritis
Source: Adv Sci (Weinh). 2025 Sep 23;13(9):e13170. doi: 10.1002/advs.202413170 (PMC12904049; doi:10.1002/advs.202413170)
Supplement: Supplementary file 1 — Supporting Information [file ADVS-13-e13170-s002.docx]

**Multiomics Analyses Reveal an Essential Role of Tryptophan in Treatment of csDMARDs in Rheumatoid Arthritis**

**Authors:** Congcong Jian^1, 17, 18*^, Jing Zhu^2*^, Jianhong Wu^3*^, Yan Zhang^4*^, Jianghua Chen^5^, Huan Wang^6^, Hengyan Liu^7^, Ke Xu^8^, Jiaxin Huang^9^, Xiaoting Zhu^1^, Yuanli Wei^3^, Shilin Li^1^, Tingting Wang^3^, Xuan Huang^10^, Qinghua Zou^11^, Jie Zhang^1^, Jiang Su^2^, Xinming Du^12^, Yaping Lu^13, 14^, Tianci Zhou^15^, Yingtong Zhou^9^, Minglong Tang^6^, Bin Li^13^, Xue Zhou^14^, Qihao Wei^14^, Qiulong, Yan^16^, Fanxin Zeng^1, 17, 18#^


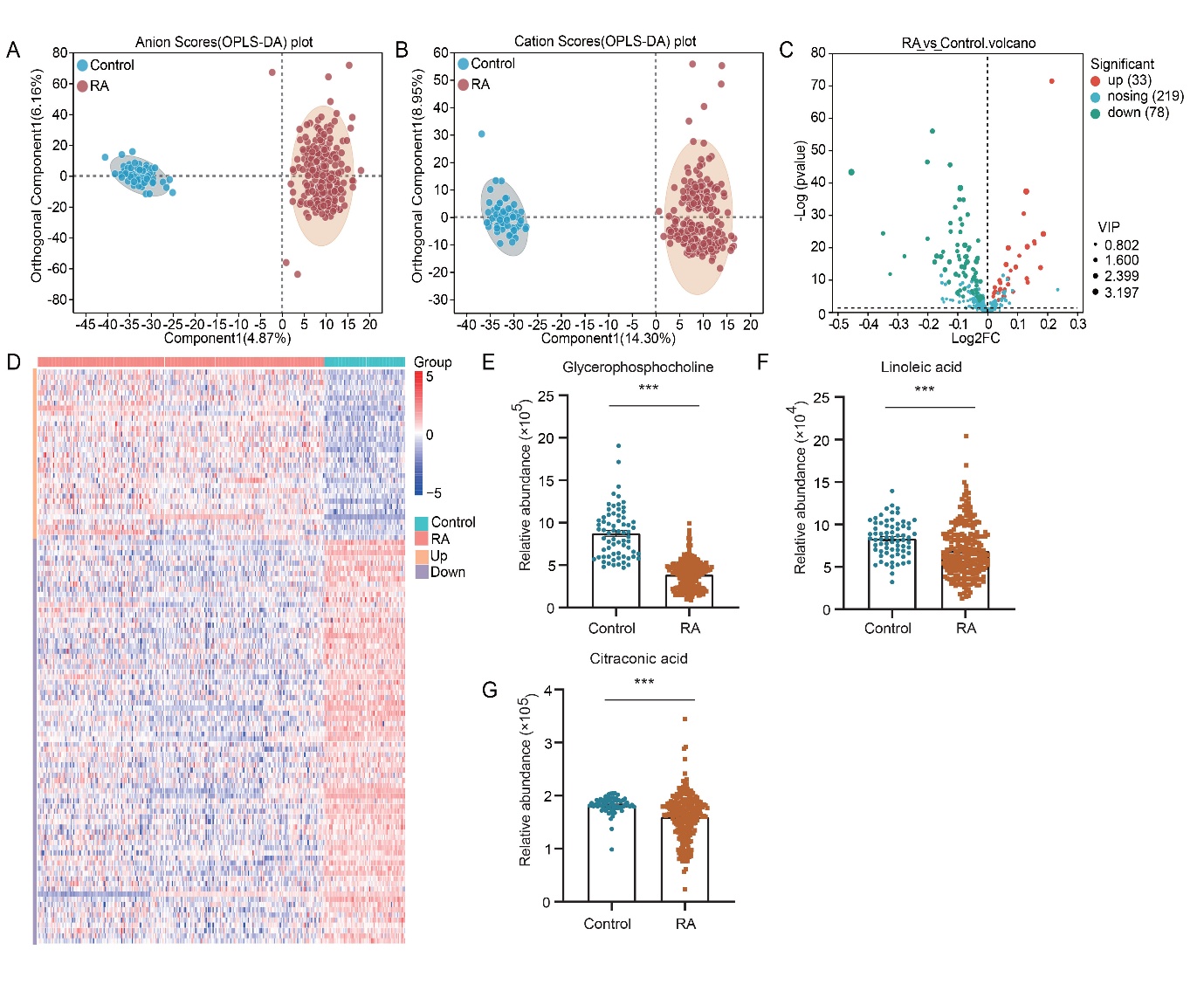


**Supplementary Figure 1. The significant alterations of plasma metabolic profile between RA patients and controls.** (A-B) A clear separation of the differential metabolites in both anionic and cationic modes between RA and controls through orthogonal partial least squares discriminant analysis (OPLS-DA), respectively. (C) The volcano plot showed the 111 significant metabolites with 33 upregulated and 78 downregulated metabolites between controls and RA patients. (D) The cluster heatmap of 111 differential metabolites were apparently distinct between RA patients and controls. (E-G) The relative abundance of Glycerophosphocholine, Linoleic acid and Citraconic acid among RA patients and controls. (RA, n=244, Control, n=69). ****, p<0.001.* Data was expressed as mean±standard error. RA, rheumatoid arthritis.


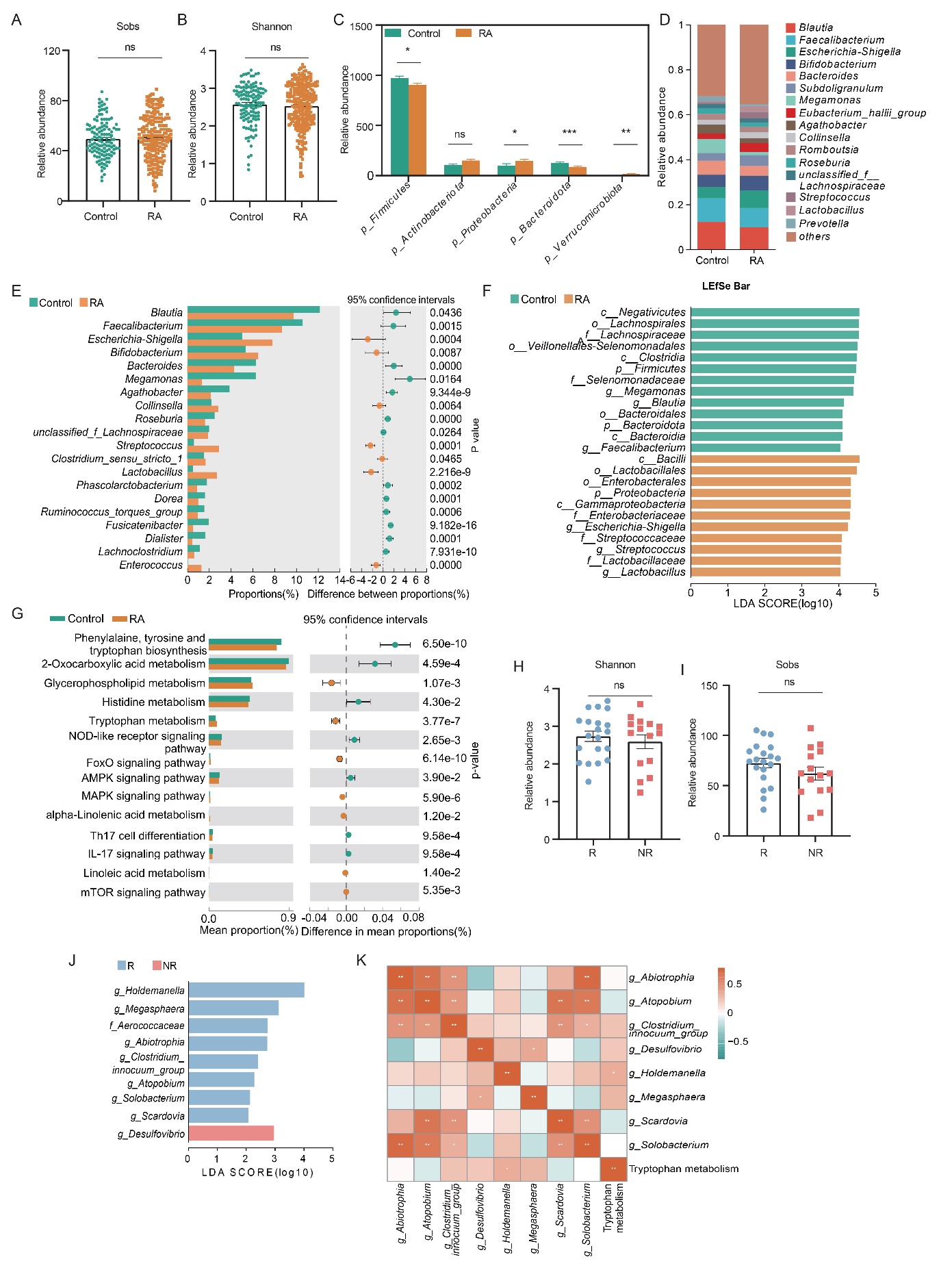


**Supplementary Figure 2. Obvious disorders in composition and community structure of gut microbiota between RA patients and controls.** (A-B) The relative abundance of sobs and shannon indexes between RA patients and controls. (C) The relative abundance of *Fimicutes, Actinobacteriota, Proteobacteria, Bacteroidota*, and *Verrucomicrobiota* at phylum level between RA patients and controls. (D) The community composition of gut microbiota at genus level between RA patients and controls. (E) The top 20 of differential bacteria between RA patients and controls by using Wilcox rank-sum test at genus level. (F) The linear discriminant analysis (LDA) showed that the important bacteria at phylum to genus level in RA patients and controls. (G) The functional predictive analysis of gut microbiota through PICRUSt2 approach. (H-I) The relative abundance of shannon and sobs indexes between two groups. (J) The linear discriminant analysis (LDA) showed that the important bacteria at phylum to genus level in response and nonresponse groups. (K) Heatmap of the correlation between 8 differential bacteria and tryptophan metabolism pathway. (A-G, RA, n=246, Control, n=124; H-K, R, n=20, NR, n=15). **, p<0.05; **, p<0.01; ***, p<0.001; ns, not significant.* Data was expressed as mean±standard error. RA, rheumatoid arthritis; R, Response; NR, Nonresponse.


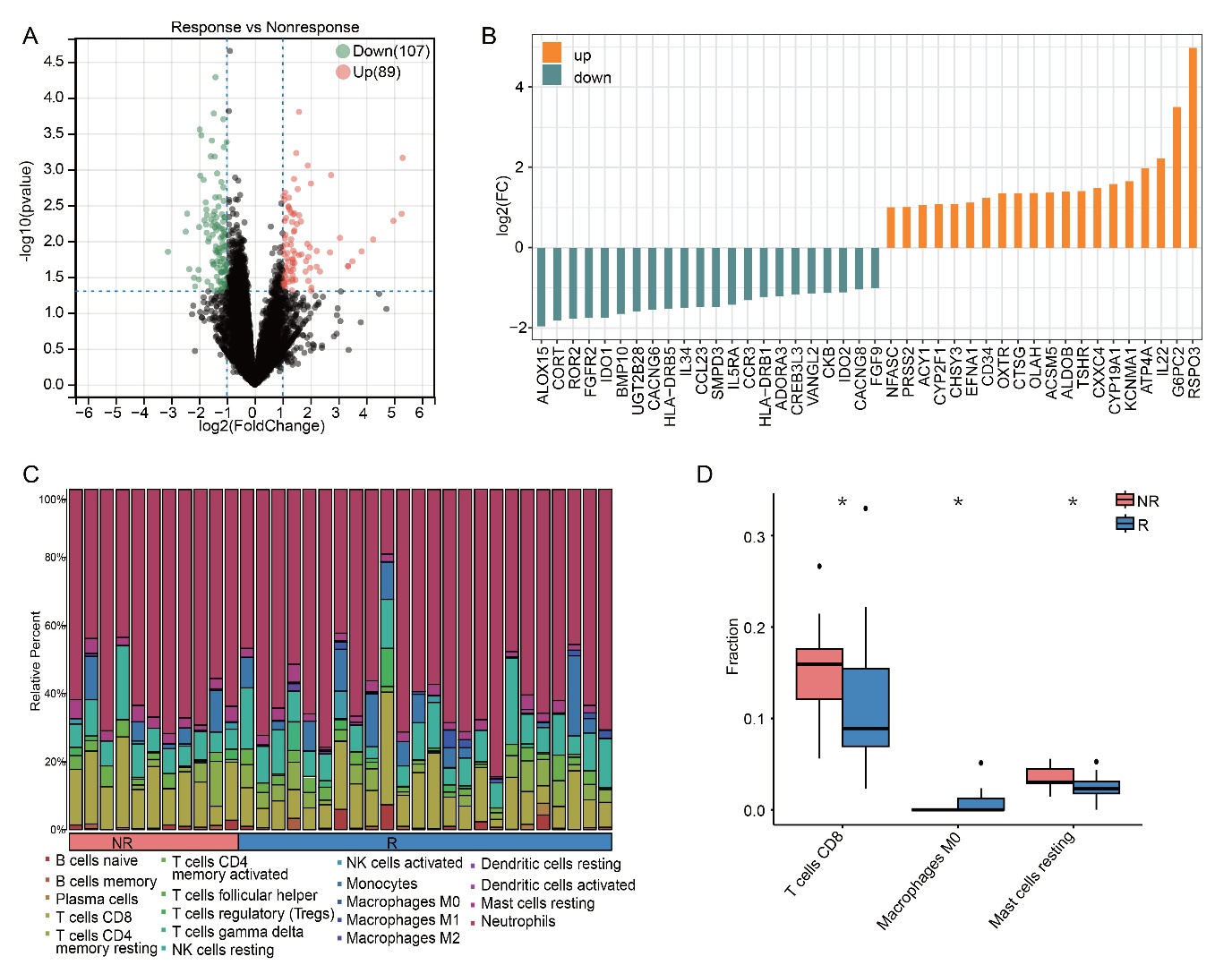


**Supplementary Figure 3. Dysregulation of gene expressed profile between response and nonresponse groups.** (A) Volcano plot showed the 196 differentially expressed genes with 89 upregulated and 107 downregulated in two groups. (B) The fold changes of 42 significant genes in 14 KEGG pathways. (C-D) The alterations of immune cells with immune cell infiltration analysis through cibersort approach. (R, n=24, NR, n=11). **, p<0.05; ns, not significant.* R, Response; NR, Nonresponse.


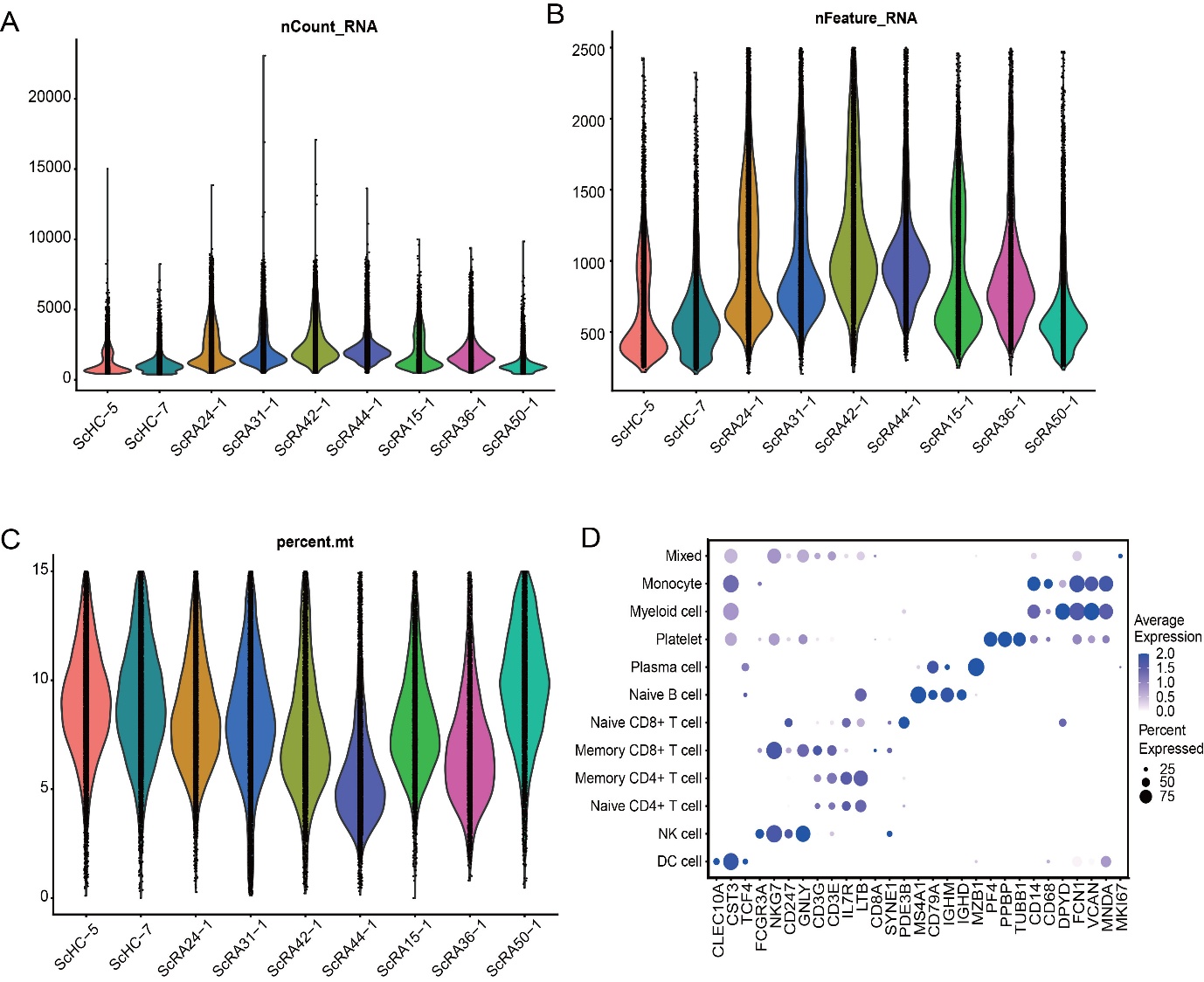


**Supplementary Figure 4. Imbalance of immune cells and immune signaling pathway among control, response and nonresponse groups.** (A-C) The data processing of single-cell transcriptomics data based on nCount_RNA, nFeature_RNA, and percent.mt in three groups. (D) The heatmap of the marker genes of 12 cell types. (E) The fraction of 12 cell types in control, response, and nonresponse groups.


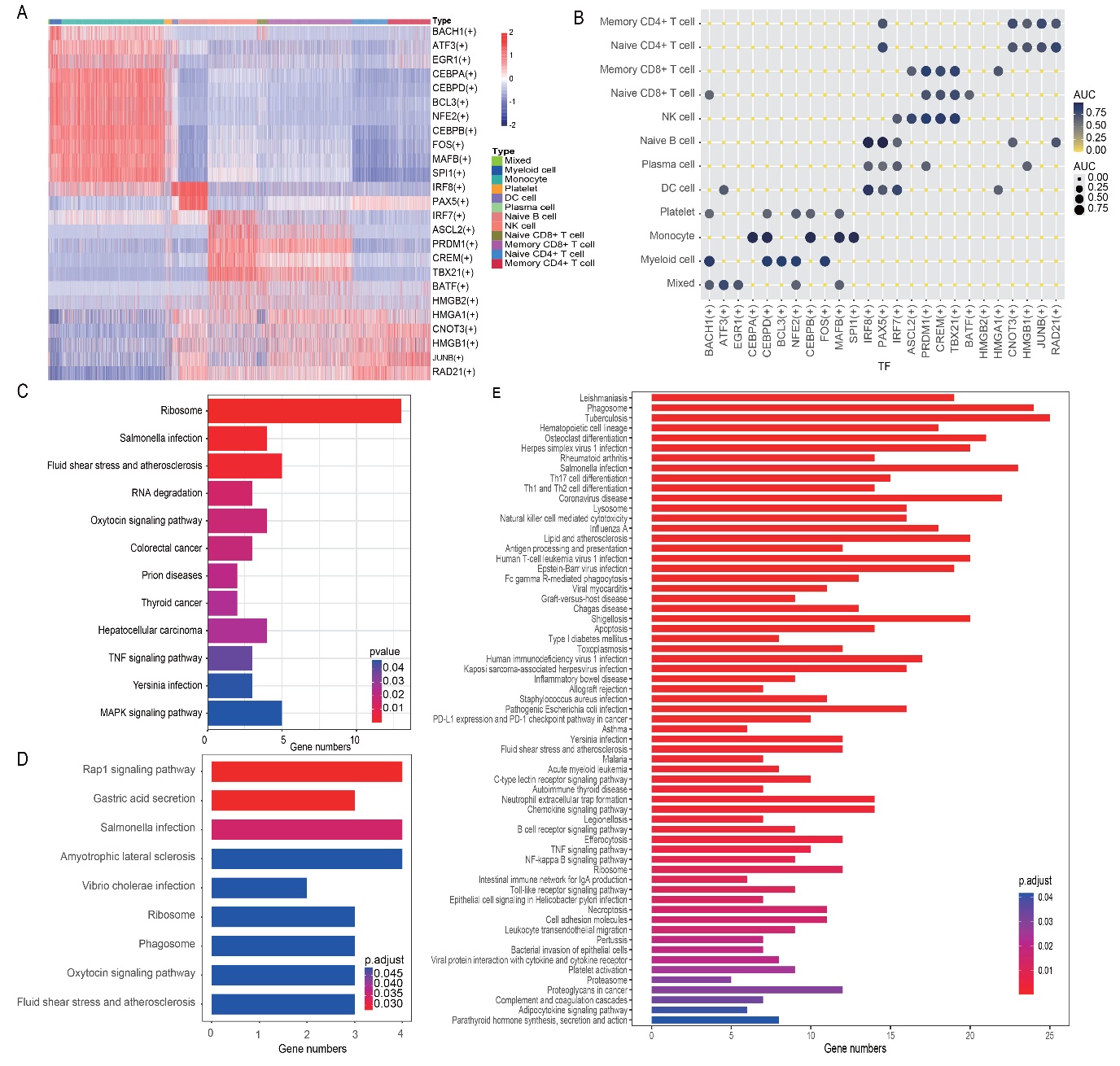


**Supplementary Figure 5. Utilizing SCENIC analysis of gene regulatory networks to identify key regulatory transcription factors.** (A) The heatmap illustrated the activity of 25 distinct transcription factors across different cell types. (B) The AUC values of marker transcription factors for each cell type. (C) KEGG enrichment analysis of target genes corresponding to transcription factors in CD4+ T cells. (D) KEGG enrichment analysis of target genes corresponding to transcription factors in CD8+ T cells. (E) KEGG enrichment analysis of target genes corresponding to transcription factors in Monocytes.


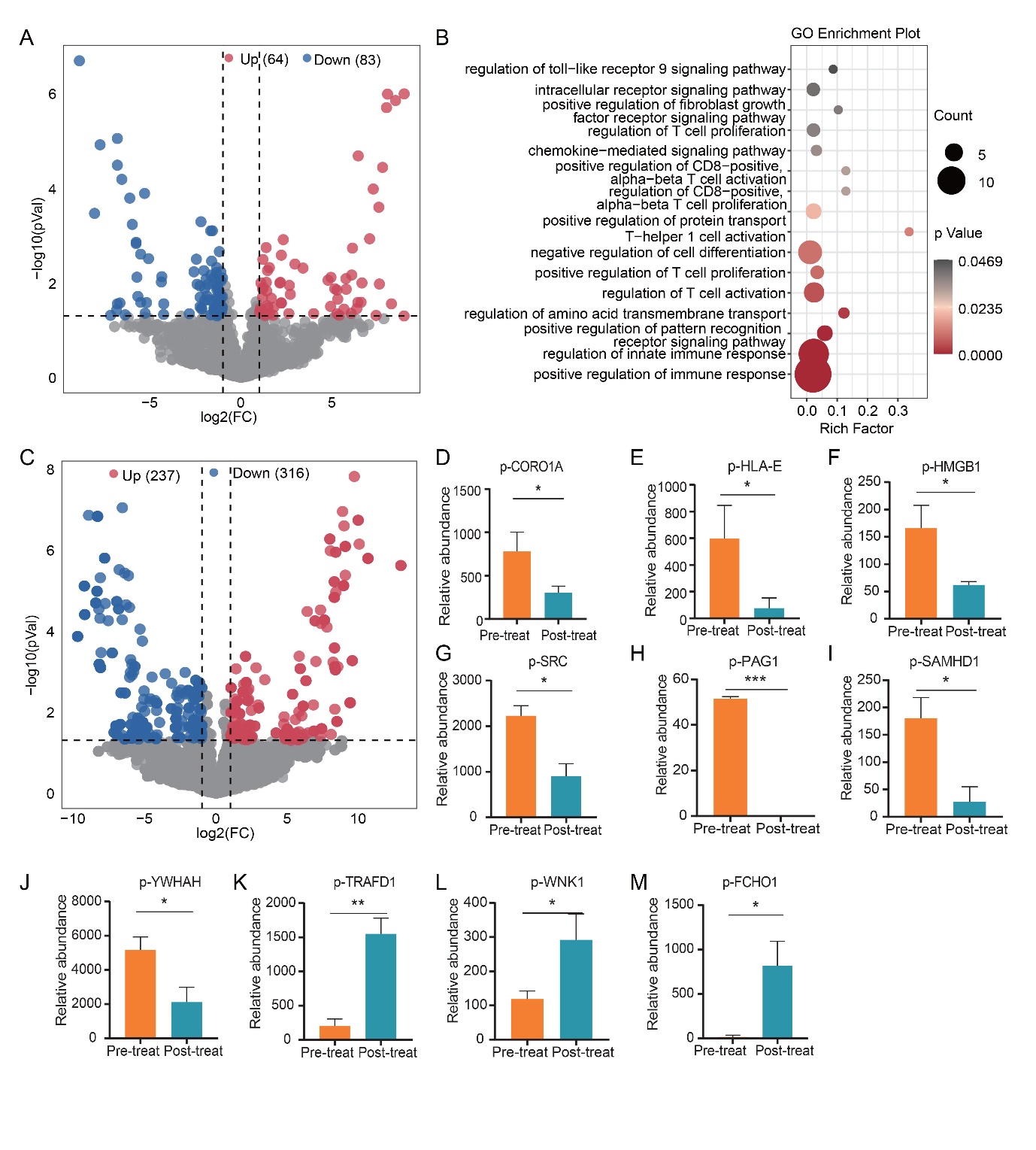


**Supplementary Figure 6. Significant changes in the immune regulatory system of phosphoproteomic profiles between pre-treat and post-treat groups.** (A) Volcano plot displayed the 147 differentially expressed phosphorylated proteins in two groups. (B) The GO enrichment of 147 significant phosphorylated proteins between pre-treat and post-treat groups. (C) The volcano plot exhibited the 553 differentially expressed phosphorylated sites between the two groups. (D-M) The expressions of phosphorylated proteins between pre-treat and post-treat groups. (Pre-treat, n=3, Post-treat, n=3). **, p<0.05; **, p<0.01.* Data was expressed as mean±standard error.


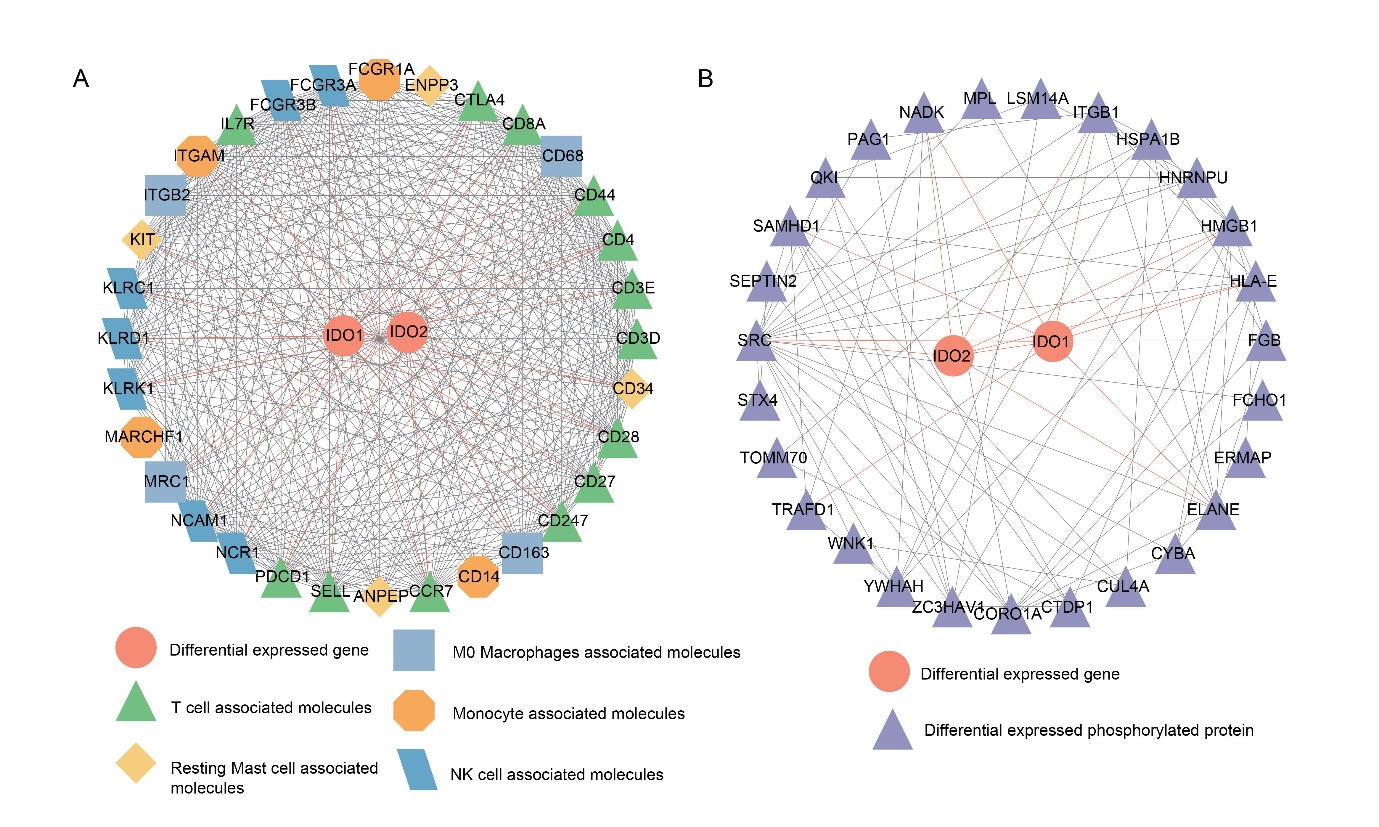


**Supplementary Figure 7.** **Interaction network diagram of key genes with immune cells related molecules and phosphorylated proteins.** (A) Protein-protein interaction network diagram of IDO1, IDO2 and immune cells related molecules. (B) Protein-protein interaction network diagram of IDO1, IDO2 and i differentially expressed phosphorylated proteins.

**
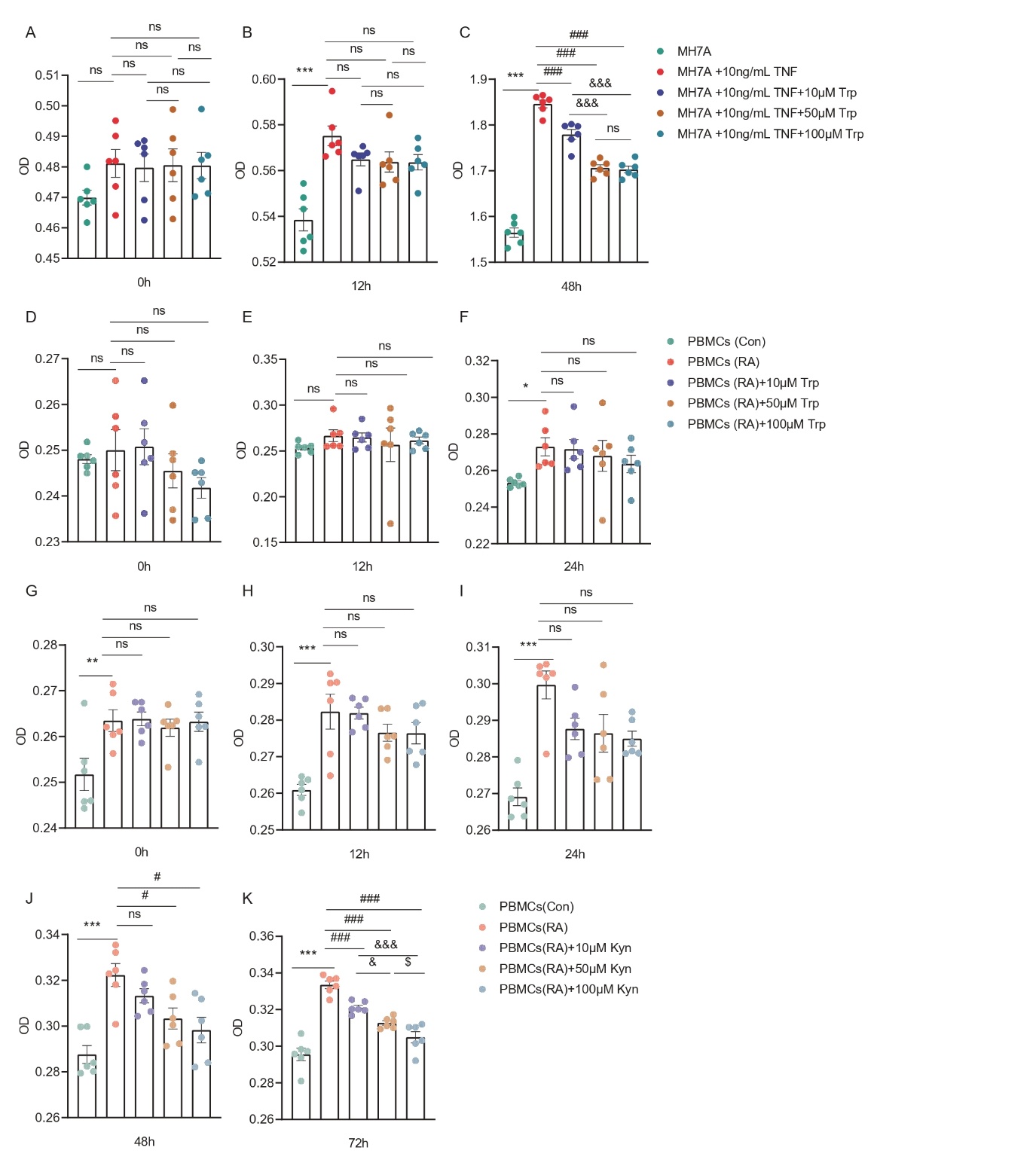
**

**Supplementary Figure 8. Cell proliferation capacity was assessed by the CCK-8 assays in varying concentrations of tryptophan and kynurenine.** (A-C) The proliferative capacity of MH7A synovial cells after 0, 12, 48 hours of intervention with varying concentrations of Trp. (D-F) The proliferative capacity of PBMCs after 0, 12, 24 hours of intervention with varying concentrations of Trp. (G-K) The proliferative capacity of PBMCs after 0, 12, 24, 48, 72 hours of intervention with varying concentrations of Kyn.


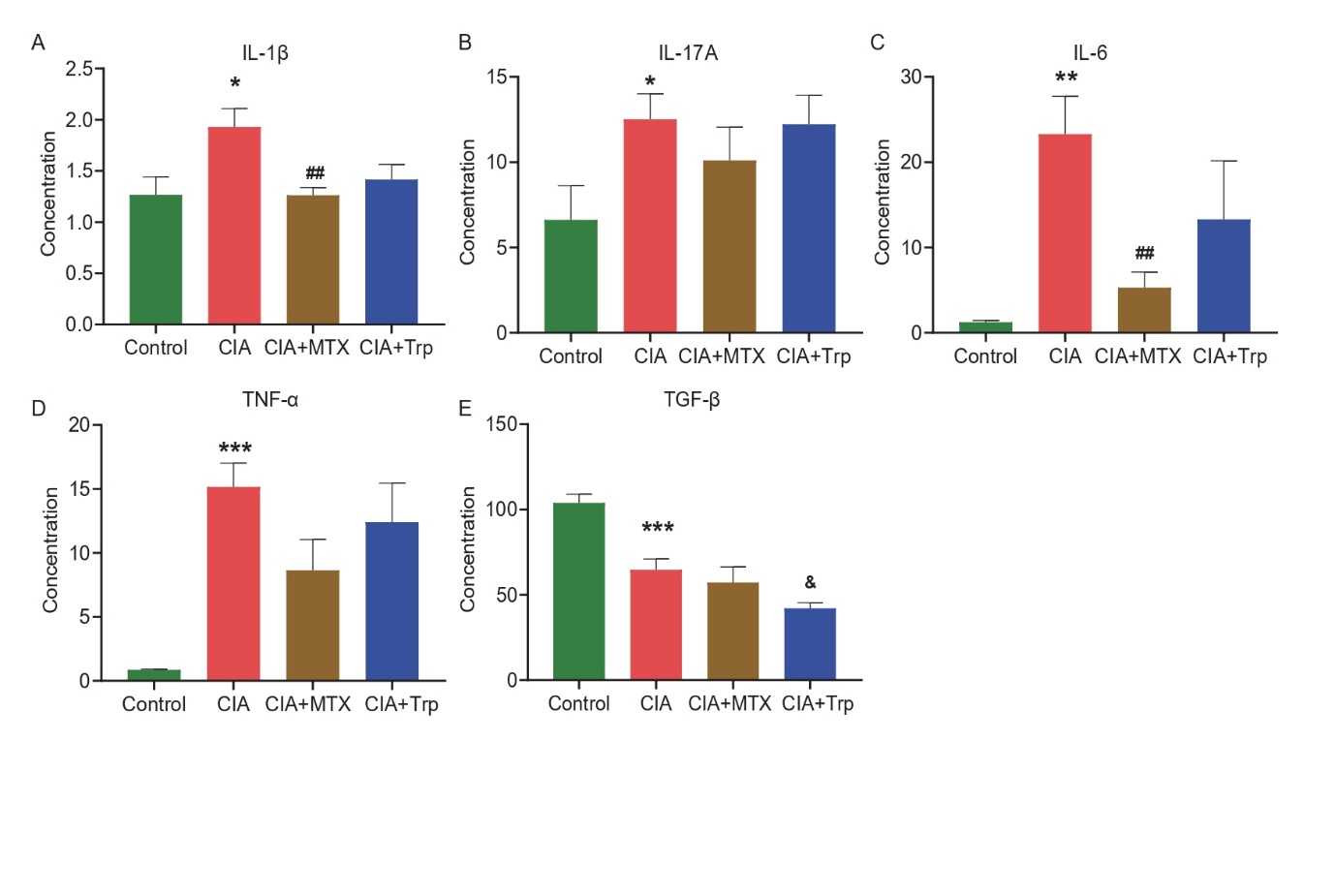


**Supplementary Figure 9. Tryptophan improved inflammatory status in mice.** (A-E) The expressions of IL-1β, IL-17A, IL-6, TNF-α, and TGF-β in serum samples of mice. Control=5, CIA=7, CIA+MTX=6, CIA+Trp=5, data was expressed as mean±standard error. * represented control group versus CIA group; # represented MTX group versus CIA group; & represented Trp group versus CIA group. **, p<0.05; **, p<0.01; ***, p<0.001. ##, p<0.01. &, p<0.05.* Data was expressed as mean±standard error. CIA, collagen-induced arthritis; MTX, methotrexate; Trp, tryptophan; IL-1β, interleukin-1beta; IL-17A, interleukin-17A; IL 6, interleukin 6; TNF-α, tumor necrosis factor alpha; TGF-β, transforming growth factor beta.

**
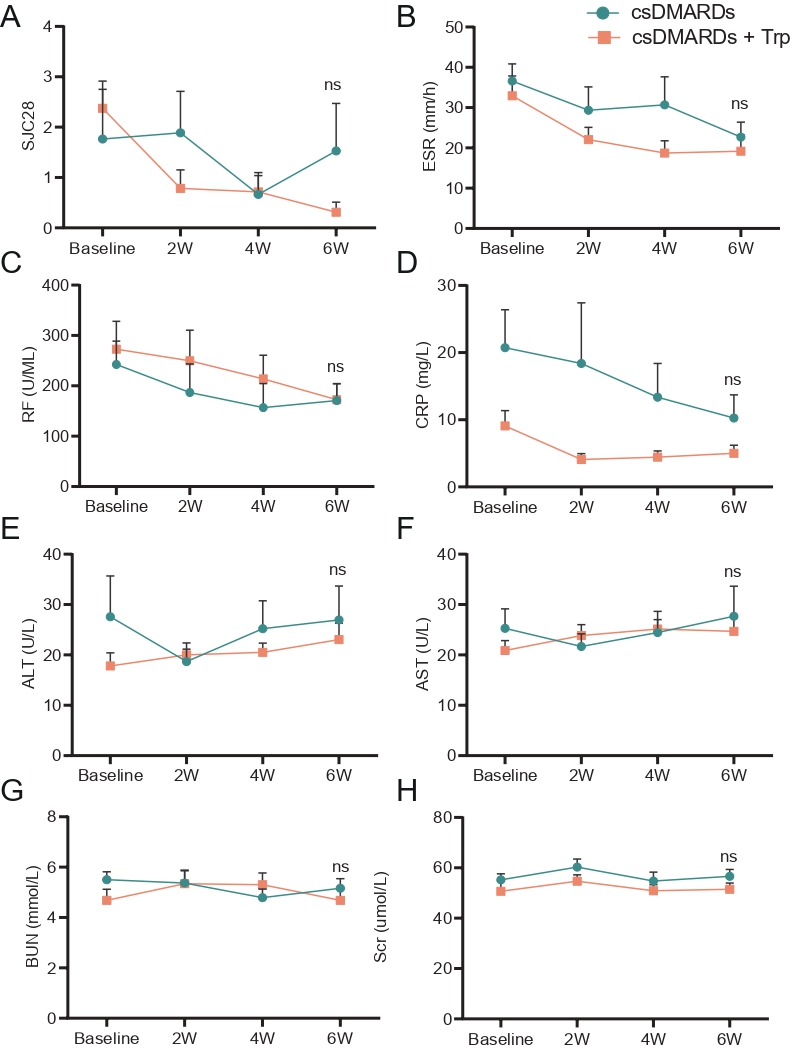
**

**Supplementary Figure 10. Dietary supplementation with tryptophan improved inflammatory status in RA patients.** (A-D) The changes of SJC28, ESR, RF, and CRP in baseline, 2w, 4w, and 6w between csMDARDs and csDMARDs+Trp groups. (E-H) The changes of ALT, AST, BUN, and Scr in baseline, 2w, 4w, and 6w between csMDARDs and csDMARDs+Trp groups. csDMARDs=17, csDMARDs+Trp=16, *ns, not significant.* Data was expressed as mean±standard error. csDMARDs, conventional synthesis disease modifying anti-rheumatic drugs; SJC, swellen joint count; ESR, erythrocyte sedimentation rate; RF, rheumatoid factor; CRP, C-reactive protein; ALT, alanine aminotransferase; AST, aspartate aminotransferase; BUN, blood urea nitrogen; Scr, serum creatinine.
