# Supplementary material for: Multiomics Analyses Reveal an Essential Role of Tryptophan in Treatment of csDMARDs in Rheumatoid Arthritis
Source: Adv Sci (Weinh). 2025 Sep 23;13(9):e13170. doi: 10.1002/advs.202413170 (PMC12904049; doi:10.1002/advs.202413170)
Supplement: Supplementary file 2 — Supporting Information [file ADVS-13-e13170-s003.pdf]

Supplementary Table 1. Clinical characteristics of the subjects in this study.

		RA (n=371)	Control (n=195)
Age (year)		58.01±12.10	49.66±8.94
Sex	Male	98	133
	Female	273	62
Duration (month)		95.17±107.13	-
SJC28		7.84±8.29	-
TJC28		9.75±8.93	-
DAS28		5.22±1.65	-
ESR (mm/h)		64.65±31.62	-
CRP (mg/L)		42.68±42.21	-
RF (U/ML)		293.65±326.94	-
IL 6 (pg/ml)		72.42±96.77	-
Medication	Methotrexate	317	-
	Leflunomide	257	-
	Hydroxychloroquine	61	-
	Iguratimod	14	-
	NSAID	142	-
	Glucocorticoids	230	-

Abbreviation: SJC, swollen joint count; TJC, tender joint count; DAS, disease activity score; ESR, erythrocyte sedimentation rate; CRP, C-reactive protein; RF, rheumatoid factor; IL 6, interleukin 6; NSAID, non-steroidal anti-inflammatory drug. Data represented as mean ± standard deviation.
