# Supplementary material for: Multiomics Analyses Reveal an Essential Role of Tryptophan in Treatment of csDMARDs in Rheumatoid Arthritis
Source: Adv Sci (Weinh). 2025 Sep 23;13(9):e13170. doi: 10.1002/advs.202413170 (PMC12904049; doi:10.1002/advs.202413170)
Supplement: Supplementary file 3 — Supporting Information [file ADVS-13-e13170-s004.pdf]

Supplementary Table 2. Clinical characteristics of responsers and nonresponsers in this study.

		Responser (n=55)	Nonresponser (n=35)	P
Age (year)		54.31±10.27	55.10±13.24	0.562
Sex	Male	14	10	0.744
	Female	41	25	
Duration (month)		61.87±81.25	95.11±108.53	0.072
SJC28		7.29±7.44	4.47±5.30	0.061
TJC28		9.95±8.16	7.50±6.89	0.117
DAS28		5.10±1.45	4.52±1.42	0.060
ESR (mm/h)		49.51±28.61	50.29±31.85	0.983
CRP (mg/L)		31.59±36.52	21.87±23.40	0.434
RF (U/ML)		354.24±449.10	279.11±298.62	0.745
IL 6 (pg/ml)		95.48±105.94	80.39±110.91	0.544
Medication	Methotrexate	54	33	0.784
	Leflunomide	38	24	
	Hydroxychloroquine	10	9	
	Iguratimod	6	6	
	NSAID	31	14	
	Glucocorticoids	32	18	

Abbreviation: SJC, swollen joint count; TJC, tender joint count; DAS, disease activity score; ESR, erythrocyte sedimentation rate; CRP, C-reactive protein; RF, rheumatoid factor; IL 6, interleukin 6; NSAID, non-steroidal anti-inflammatory drug.Data represented as mean ± standard deviation.
