# Supplementary material for: Multiomics Analyses Reveal an Essential Role of Tryptophan in Treatment of csDMARDs in Rheumatoid Arthritis
Source: Adv Sci (Weinh). 2025 Sep 23;13(9):e13170. doi: 10.1002/advs.202413170 (PMC12904049; doi:10.1002/advs.202413170)
Supplement: Supplementary file 4 — Supporting Information [file ADVS-13-e13170-s001.pdf]

Supplementary Table 3. Baseline of subjects included in clinical trial study.

		csDMARDs (n=17)	csDMARDs+Trp (n=16)	P
Age (year)		51.12±10.58	46.81±10.90	0.258
Sex	Male	4	3	1.000
	Female	13	13	
Duration (month)		31.93±37.55	69.75±45.79	0.004**
SJC28		1.76±4.07	2.38±2.16	0.017*
TJC28		5.29±4.51	5.13±4.16	0.913
DAS28		4.07±0.98	3.89±0.81	0.482
HAQ		1.24±1.44	1.63±2.03	0.836
VAS		1.76±0.44	1.81±0.83	0.768
ESR (mm/h)		36.59±17.66	32.94±19.60	0.578
CRP (mg/L)		20.74±23.27	9.08±9.12	0.061
RF (U/ML)		236.15±181.94	272.81±221.05	0.746
ALT (U/L)		26.60±29.49	17.81±10.46	0.526
AST (U/L)		25.67±14.04	20.88±7.92	0.198
WBC		6.90±2.61	7.04±2.60	0.930
NEUT		4.82±2.04	4.80±2.44	0.734
LY		1.50±0.72	1.67±0.75	0.749
MONO		0.45±0.14	0.49±0.16	0.955
HB (g/L)		125.00±15.06	131.00±13.50	0.338
PLT		267.33±73.94	256.06±69.58	0.576
Medication	Methotrexate	16	16	0.221
	Leflunomide	10	12	
	Hydroxychloroquine	7	3	
	Glucocorticoids	2	7	
	NSAID	8	4	

Abbreviation: csDMARDs, conventional synthesis disease modifying anti-rheumatic drugs; Trp, tryptophan; SJC, swollen joint count; TJC, tender joint count; DAS, disease activity score; HAQ, Health assessment questionnaire; VAS, visual analogue scale; ESR, erythrocyte sedimentation rate; CRP, C-reactive protein; RF, rheumatoid factor; ALT, alanine aminotransferase; AST, Aspartate aminotransferase; WBC, white blood cell; NEUT, neutrophile; LY, lymphocyte; MONO, monocyte; HB, Hemoglobin; PLT, platelet; NSAID, non-steroidal anti-inflammatory drug. Data represented as mean ± standard deviation. *, $p<0.05$, **, $p<0.01$.
